# Combined NADPH Oxidase 1 and Interleukin 10 Deficiency Induces Chronic Endoplasmic Reticulum Stress and Causes Ulcerative Colitis-Like Disease in Mice

**DOI:** 10.1371/journal.pone.0101669

**Published:** 2014-07-09

**Authors:** Xavier Tréton, Eric Pedruzzi, Cécile Guichard, Yannick Ladeiro, Shirin Sedghi, Mélissa Vallée, Neike Fernandez, Emilie Bruyère, Paul-Louis Woerther, Robert Ducroc, Nicolas Montcuquet, Jean-Noel Freund, Isabelle Van Seuningen, Frédérick Barreau, Assiya Marah, Jean-Pierre Hugot, Dominique Cazals-Hatem, Yoram Bouhnik, Fanny Daniel, Eric Ogier-Denis

**Affiliations:** 1 INSERM, UMRS1149, Team «Physiopathology of Inflammatory Bowel Diseases», Centre de Recherche sur l’Inflammation, Paris, France; 2 Université Paris-Diderot Sorbonne Paris-Cité, Paris, France; 3 Service de Gastroentérologie et d'Assistance Nutritive, PMAD Hôpital Beaujon, Clichy la Garenne, France; 4 Laboratory of Excellence Labex INFLAMEX, PRES Paris Sorbonne Cité, France; 5 INSERM, UMRS871, Centre Biomédical des Cordeliers, Paris, France; 6 INSERM, UMR837, Team 5 «Mucins, epithelial differentiation and carcinogenesis», Jean-Pierre Aubert Research Center, Lille, France; 7 Laboratoire de Bacteriologie, Hôpital Bichat-Claude Bernard, Paris, France; 8 INSERM, U989, Université Paris-Descartes, Necker, Paris, France; 9 Université Paris Descartes, Faculté de Médecine René Descartes, Paris, France; 10 INSERM, U682, Université Louis Pasteur, Strasbourg, France; 11 INSERM, U843 Hôpital R. Debré, Paris, France; 12 Service d’Anatomo-Pathologie, Hôpital Beaujon, Clichy, France; Institut Pasteur de Lille, France

## Abstract

Ulcerative colitis (UC) is a chronic inflammatory bowel disease affecting the rectum which progressively extents. Its etiology remains unknown and the number of treatments available is limited. Studies of UC patients have identified an unbalanced endoplasmic reticulum (ER) stress in the non-inflamed colonic mucosa. Animal models with impaired ER stress are sensitive to intestinal inflammation, suggesting that an unbalanced ER stress could cause inflammation. However, there are no ER stress-regulating strategies proposed in the management of UC partly because of the lack of relevant preclinical model mimicking the disease. Here we generated the IL10/Nox1^dKO^ mouse model which combines immune dysfunction (IL-10 deficiency) and abnormal epithelium (NADPH oxidase 1 (Nox1) deficiency) and spontaneously develops a UC-like phenotype with similar complications (colorectal cancer) than UC. Our data identified an unanticipated combined role of IL10 and Nox1 in the fine-tuning of ER stress responses in goblet cells. As in humans, the ER stress was unbalanced in mice with decreased eIF2α phosphorylation preceding inflammation. In IL10/Nox1^dKO^ mice, salubrinal preserved eIF2α phosphorylation through inhibition of the regulatory subunit of the protein phosphatase 1 PP1R15A/GADD34 and prevented colitis. Thus, this new experimental model highlighted the central role of epithelial ER stress abnormalities in the development of colitis and defined the defective eIF2α pathway as a key pathophysiological target for UC. Therefore, specific regulators able to restore the defective eIF2α pathway could lead to the molecular remission needed to treat UC.

## Introduction

Ulcerative colitis (UC) is the most common chronic inflammatory disorder affecting exclusively the colon [1]. UC is a complex disease due to deregulated interactions between epithelial cells, immune and environmental factors. UC is mainly characterized by: 1) universal rectal involvement with upstream colonic lesions, 2) superficial colonic mucosal inflammatory damage, 3) early goblet cell alterations even in non-inflamed colonic tissues, 4) polymorphonuclear infiltrates and crypt abscesses at the acute inflammatory stage, 5) disease onset and outcome prevented by tobacco smoking and appendicitis at a young age and 6) long-term increased risk of developing colonic dysplasia/cancer.

A growing body of evidence suggests that the colonic epithelial homeostasis could be a critical element mediating protection from detrimental environmental factors and regulating underlying inflammatory responses in UC [2], [3], [4], [5]. Colonic epithelial cells, and especially goblet cells whose secretory functions depend on protein synthesis, have developed evolved mechanisms to cope with cellular stresses such as the ER stress and inflammation. It is now evident that an unresolved ER stress in intestinal epithelial cells associated with altered unfolded protein response (UPR) activation, a process induced by three ER proximal sensors PERK, ATF6, and IRE1 [6], can lead to or induce a sensitivity to colonic inflammation both in animals [3], [4], [7], [8], [9], [10], [11] and humans [4], [5]. Paradoxically, partial or total goblet cell depletion does not cause spontaneous colitis [12], [13] and can even reduce dextran sodium sulfate-induced colonic injury [13] suggesting that the predisposition to colitis might be promoted in the goblet cells themselves due to their inability to provide protection against environmental factors. Recently, IL-10 has been shown to play a central role in goblet cell homeostasis by suppressing the ER stress and promoting intestinal mucus production [2], [9], [14]. Moreover, IL-10 polymorphisms and rarer mutations in the *IL-1*0 and *IL-10R* genes have been associated with inflammatory bowel diseases (IBD) [15], [16] and severe enterocolitis in infants [17], respectively. Furthermore, mice lacking IL-10 spontaneously develop colon and ileum inflammation similar to Crohn’s disease [18]. The current paradigm for the regulatory roles of IL-10 in epithelial cell homeostasis [19] and ER stress response in goblet cells [2], [14] reinforces the central role of the epithelial barrier in UC pathogenesis.

We have previously shown that the NADPH oxidase 1 (Nox1), a reactive oxygen species (ROS)-producing oxidase highly expressed in colonic epithelial cells, controls the balance between goblet and absorptive cells in the colon by coordinately modulating the PI3K/AKT/Wnt/beta-catenin and Notch1 signaling pathways [20]. Nox1-deficient (Nox1^KO^) mice show a massive conversion of progenitor cells into functional goblet cells without developing any colitis [20]. A growing body of evidence indicates close functional links between Nox1 and intestinal epithelial cells. Jones *et al.* [21] have recently shown that the commensal *Lactobacillus* spp. can stimulate Nox1-dependent ROS production and subsequent intestinal stem cell proliferation, highlighting the important role of Nox1 in critical ROS-mediated colonic homeostatic processes. Furthermore, Leoni *et al*. [22] have shown the central role of intestinal epithelial Nox1 in facilitating ROS-dependent mucosal epithelial wound repair mediated by gut microbiota-induced N-formyl peptide receptors [22]. Importantly, ROS production by Nox enzymes is critical to control the mucin granule accumulation and release in colonic goblet cells [23]. Moreover, NOX1 expression follows the same colonic gradient than the thickness of the mucus layer secreted by goblet cells [24] and UC lesions. Taken together, these findings suggest that a suitable UC model would show goblet cell accumulation and would be highly susceptible to inflammation. For these reasons, we combined *Nox1* and *IL-10* gene deletion to generate double knockout (IL10/Nox1^dKO^) mice.

We showed that IL10/Nox1^dKO^ mice spontaneously reproduced all the clinical and biological features of human UC and exhibited similar ER stress alterations than those observed in UC patients, including a dramatic loss in eIF2α phosphorylation and goblet cells, highlighting the importance of this pathway in the onset of colitis. Mechanistically, we demonstrated that IL10 and Nox1 were two major negative regulators of the ER stress in goblet cells and helped preserving the colonic mucus barrier. Furthermore, this experimental mouse model of UC provides a unique opportunity to test, at a preclinical level, pharmacological interventions which prevent eIF2α dephosphorylation, and develop new drugs targeting the colonic epithelium in UC.

## Methods

### Recruitment of human participants

12 healthy controls and 12 patients with UC were recruited at the IBD Gastroenterology Unit, Hôpital Beaujon (see Table S1). The protocol was approved by the local Ethics Committee (CPP-Ile de France IV No. 2009/17) and written informed consent was obtained from all patients before enrollment. Non-inflamed colonic areas were biopsied during colonoscopy procedure in all patients with UC and in healthy controls. One biopsy was analyzed histologically to assess the absence of colitis, five biopsies were snap frozen and then stored in liquid nitrogen for molecular analysis, and three biopsies were fixed in glutaraldehyde for electron microscopy.

### Mice

C57BL/6-WT and C57BL/6-IL10^KO^ (Charles River Laboratories), and C57BL/6-Nox1^KO^ mice (kindly provided by Pr. K.H. Krause, Geneva Switzerland) were bred and housed under Specific Pathogen Free (SPF) conditions. Nox1^KO^ mice have been described previously [20] and were crossed with IL10^KO^ mice to generate C57BL/6-IL10/Nox1^dKO^ mice. A Mendelian ratio of IL10/Nox1^dKO^ offspring were born and developed normally. The only gender difference observed was an earlier onset of colitis in males. Therefore, we only used males in our experiments. All mouse experiments were approved by the local Animal Ethics Review Committee of the Faculty/Paris 7 University. Blood sera from sentinel mice were tested for pathogens and a number of viruses.

### Clinical Assessment of colitis

The disease activity index (DAI) score was assessed from 3 weeks of age. Mice were assessed for changes in weight, stool frequency and consistency, presence of blood in stools, length-to-weight colon ratio, and prolapsed rectum.

### Histological grading

Mouse colons were collected, flushed with cold PBS, cut into small pieces (proximal, median, and distal segments) or open longitudinally, fixed with 10% formalin (Sigma-Aldrich), and then embedded in paraffin as “Swiss rolls” containing the full-length organ. Small intestines were also fixed. Paraffin-embedded sections (5 µm) were deparaffinized and stained with H&E and AB/PAS reagents.

Histological grading of colitis was performed on the distal ileum and the proximal, median and distal colon by scoring the leukocyte infiltration (score range 0–3), neutrophil infiltration (0–3), crypt abscesses (0–3), epithelial damages and ulcerations (0–3), goblet cell depletion (0–2), aberrant crypt architecture (0–3), and mucosal hyperplasia (0–2). Histological scoring was performed blindly by a pathologist expert in intestinal inflammation (DCH).

### Treatments

WT and Nox1^KO^ mice were treated with 4% DSS (Mw 40 kDa, MP Biomedicals) administered through the drinking water for 5 consecutive days or with a rectal enema of 0.85 mmol/kg TNBS (Sigma) (60% H_2_0/40% ethanol, v/v). TNBS-treated mice and ethanol-treated control mice were sacrificed 24 h later. WT and Nox1^KO^ mice were treated intraperitoneally with 2 µg/g tunicamycin (Sigma). Animals were sacrificed 24 h later. For the assessment of the clinical score of DSS-, TNBS- and tunicamycin-treated mice, prolapses were excluded from the DAI.

Three-4-week old IL10/Nox1^dKO^ mice received intraperitoneally 1 mg/kg salubrinal (Calbiochem) 3 days/week for 3 weeks and were then sacrificed.

The DAI and histological colitis scores were assessed as described above.

### Immunohistochemistry and immunofluorescence

Immunohistochemistry was performed as described previously [20] using antibodies directed against phospho-histone 3 (Upstate), PCNA and GRP78/Bip (Abcam), cleaved caspase-3 (Cell Signaling), Muc2, and ATF6α (Santa Cruz Biotechnology, Tebu-bio), phospho-eIF2α (Cell Signaling), Foxp3 (eBiosciences), and Muc4 [25].

Immunofluorescence studies were performed using antibodies directed against KDEL (Enzo Life Sciences), GRP78/Bip and active caspase 3 (Cell Signaling), and Muc2 (Santacruz), and then labeled with the appropriate secondary antibody (Life Technologies). Nuclei were stained using TO-PRO-3 iodide. Fluorescence was detected by confocal laser scanning microscopy (CLSM-510-META, Zeiss). All images were acquired using the Zeiss LSM Image Browser software.

### Chimeric mice

Bone marrow stem cells (BM) were isolated from WT, IL10^KO^ or IL10/Nox1^dKO^ CD45.2/Ly5.2 mice. Five million BM were injected intravenously into WT CD45.1/Ly5.1 lethally-irradiated recipients (900 cGy of ionizing radiation) and the chimerism was assessed at week 16 by flow cytometry using Ly5.1 and Ly5.2 markers. About 85–90% of immune cells were derived from the grafted bone marrow: at least 85% of T-cells, 95% of B-cells and 85% of DC found in spleens were from the donor.

### Preparation of cell suspensions from the spleen

Briefly, the spleen was removed from mice and washed with cold PBS. Cell suspensions were prepared by extracting the cells with a 5-ml polypropylene syringe piston. The cells were centrifuged, erythrocytes were lysed by addition of Gey’s-solution, and suspended in PBS.

### Lymphocyte isolation

Colonic lamina propria mononuclear cells were isolated from WT (n = 5) and IL10/Nox1^dKO^ mice (n = 5) and aliquots of the leukocyte fractions were prepared for flow cytometric analysis as described by Schulthess et al. [26].

### Flow cytometry analysis

Cell suspensions were incubated with PE-, FITC-, APC-, or PerCP-conjugated mAbs against mouse CD3, CD4, CD8, CD11c, CD19, CD44^high^, CD62^l^°^w^, NK1, CD45Ly.1, and CD45Ly.2 (BD Biosciences) at optimal concentrations for 20 minutes at 4°C. Antibodies for the intracellular staining of Foxp3+ Tr_eg_ cells were from eBioscience. Labeled cells were analyzed using a BD-LSR II device and CELLQuest software (BD Biosciences).

### Cell culture and treatments

HT-29Cl16E cells (Ephyscience) were cultured in Dulbecco's modified Eagle's medium (4.5 g/liter glucose) supplemented with glutamax, 10% calf serum, 100 µg/ml streptomycin, and 100 U/ml penicillin at 37°C in a 5%-CO_2_ environment as previously described (38). Nox1 stealth RNAi siRNA or stealth RNAi siRNA negative control Med GC (Life Technologies) was transfected into cells using Lipofectamine ™ RNAimax reagent (Life Technologies). Cells were maintained in the same medium for 48 hours. Twenty-four hours before cell harvesting, human recombinant IL-10 (rhIL-10, 50 ng/ml, R&D System) was added to the medium. Cells were treated with TM (5 µg/ml), Tg (5 µM, Sigma-Aldrich) or DMSO 6 hours before harvesting. Cell supernatants were collected 48 hours after siRNA transfection. IL-8 expression was measured by ELISA using the BD OptEIA kit (BD Biosciences) according to the manufacturer’s instructions.

### Proximity ligation assay

HT-29Cl16E cells (24,000 cells) were plated on 6 channel µ-Slide VI 0.4 (Ibidi) then fixed and the proximity ligation assay (PLA) was carried out according to the manufacturer’s instructions (Olink Biosciences). Briefly, fixed cells were permeabilized with 0.2%Triton X100 and incubated in blocking solution for 30 min before adding the following primary antibodies overnight at 4°C: mouse anti-PP1c and rabbit anti-GADD34 antibodies (1/100 dilution). After washing, the anti-rabbit MINUS and anti-mouse PLUS PLA probes were added at 1/5 in blocking solution for 1 h at room temperature. Ligation was carried out for 30 min at 37°C and amplification was done according to the manufacturer’s protocol. The concatemeric amplification products extending from the oligonucleotide arm of the PLA probes were then detected using a confocal scanning microscope.

PLA signals were quantified from at least 8 images. High-resolution images from single scans were analyzed with the Imaris 7.7 software from BitPlane to calculate the density of PLA puncta per cell. Images were first smoothed and a threshold was selected to discriminate PLA puncta from background fluorescence. Once selected, this threshold was applied uniformly to all images in the sample set.

### Detection of phospho-eIF2α using an alphascreen SureFire assay

HT-29Cl16E cells (60,000 cells/well) carrying scrambled or Nox1 siRNA were seeded into 96-well culture microplates in a volume of 50 µl. After resting, the cells were treated with 50 ng/ml IL10 for 24 hours, 5 µg/ml TM for 4 hours or with a combination of IL10+TM and then lysed in Lysing buffer according to the manufacturer’s protocol (Perkin Elmer). A portion of lysate from each well (4 µl) was transferred to a 384-well ProxiPlate, and assayed for phospho-eIF2α. A mixture of Reaction buffer, Activation buffer, and AlphaScreen Acceptor beads was prepared according to the manufacturer’s instructions, and 5 µl of the assay mixture was added to the lysate in each well. The plates were sealed and covered in foil, and incubated at 22°C for 2 h. Then a mixture of 2 µl Dilution buffer and AlphaScreen donor beads were added in the wells. The plates were sealed and covered in foil, and incubated at 22°C for 2 h. The signal in the wells was detected using an EnSpire Alpha plate reader (Perkin Elmer).

### Bacterial translocation

Mice were sacrificed and the spleen was aseptically removed. Under sterile conditions, a sample fragment was cut tangentially and sections placed on microscope slides. The remaining material was crushed in a brain-heart infusion with 10% glycerol for storage at −80°C. 50 µl of homogenate were also placed on the slides. Both sections and homogenates were examined after Gram staining. One hundred fields/slide were analyzed. The cultures were performed by plating each sample (1/100 and 1/10000 dilutions) on blood supplied agar (BioMerieux, France) and were incubated at 37°C for 48 h in aerobic and anaerobic atmospheres. The number of CFU/g of tissue was quantified. Colonies obtained underwent polymerase chain reaction (PCR) and sequencing of their 16SrRNA gene for precise specy identification [27].

### Electron Microscopy

Normal colon biopsies from controls, patients with UC, and unaffected colonic sections of 4-week old WT, Nox1^KO^, IL10^KO^, and IL10/Nox1^dKO^ mice were extemporaneously fixed in 1.7% glutaraldehyde in 0.1 M sodium cacodylate buffer (pH 7.2) for 24 h at 4°C, post-fixed in osmium tetroxide, dehydrated in ethanol and embedded in Epon. For transmission electron micrographs analysis, ultrathin sections stained with lead citrate were examined on a Jeol 1010 electron microscope. For scanning electron microscopy, colonic samples were dried after substitution with liquid CO_2_ in a critical-point dryer (Polaron Equipment Ltd., Watford) and coated with gold (SEM coating unit E5100; Polaron). Samples were viewed with a Philips 505 SEM microscope.

### Assessment of colonic hyperproliferation

The length of mouse proximal, median, and distal colonic crypts was measured on longitudinal sections in each colonic area using a micrometer.

### Measurement of paracellular permeability

After sacrifice, biopsies of colonic mucosa were mounted in Ussing chambers and maintained in circulating oxygenated Ringer solution at 37°C throughout the experiment. Paracellular permeability was assessed by measuring the mucosal-to-serosal flux of 4 kDa FITC-dextran (Sigma, France) as previously described [28].

### Quantitative real-time PCR

Colon samples for qRT-PCR were extracted with RNAble (Eurobio) and quantified using a ND-1000 NanoDrop spectrophotometer (NanoDrop Technologies). Purity/integrity was assessed with disposable RNA chips (Agilent RNA 6000 Nano LabChip kit) using an Agilent 2100 Bioanalyzer (Agilent Technologies). Reverse transcription was performed using M-MLV (Invitrogen). Q-PCR was performed with SYBR Green using a LightCycler 480 instrument (Roche Diagnostics). Values were calculated using the Δ*Ct* method and were normalized to the housekeeping gene. Primer sequences can be provided upon request.

### Western Blot analyses

Colonic tissues were homogenized in a radioimmunoprecipitation assay buffer (50 mM Tris-Cl [pH 8.0], 320 mM sucrose, 0.1 mM EDTA, 1 mM DTT, 1% Nonidet P-40, 0.1% SDS and 1% protease/phosphatase I and II inhibitor cocktail [Sigma]). Proteins (50–100 µg) were separated on 10% SDS-PAGE gel, transferred onto a membrane using iBlotGel Transfer device (Invitrogen), and probed with primary antibodies: Phospho-eIF2α (Ser^51^) (Cell Signalling), eIF2α, ATF4, and GADD34 (Santa Cruz Biotechnology), KDEL (Abcam), and β-actin (Sigma). Horseradish peroxidase-conjugated secondary antibodies were detected using ECL reagents (Pierce).

### Enzyme-linked immunosorbent assay

Cytokines were measured by ELISA using the manufacturer’s guidelines (eBiosciences).

### Statistical analyses

Because of difficulties to confirm a normal distribution due to the sample size, statistically significant differences between the four different types of mice over time were assessed using the non-parametric Kruskal-Wallis test with Dunn’s multiple comparison test, and data are presented using box plots. The non-parametric Mann-Whitney *U*-test was used to analyze changes between two groups. All statistical analyses were performed using Prism v5.0 (Graphpad software). The statistical test used and sample sizes for individual analyses are indicated in the figure legends.

## Results

### IL10/Nox1^dKO^ mice spontaneously develop colitis with features similar to human UC

IL10/Nox1^dKO^ mice were monitored and compared to age-matched wild type (WT), IL-10^KO^, and Nox1^KO^ mice. Mice were submitted to systematic clinical analysis, comprehensive necropsy with histopathological examination of the entire alimentary tract at 3–4 weeks, 6–7 weeks, 10–12 weeks and 6–8 months of life.

**Figure 1:**
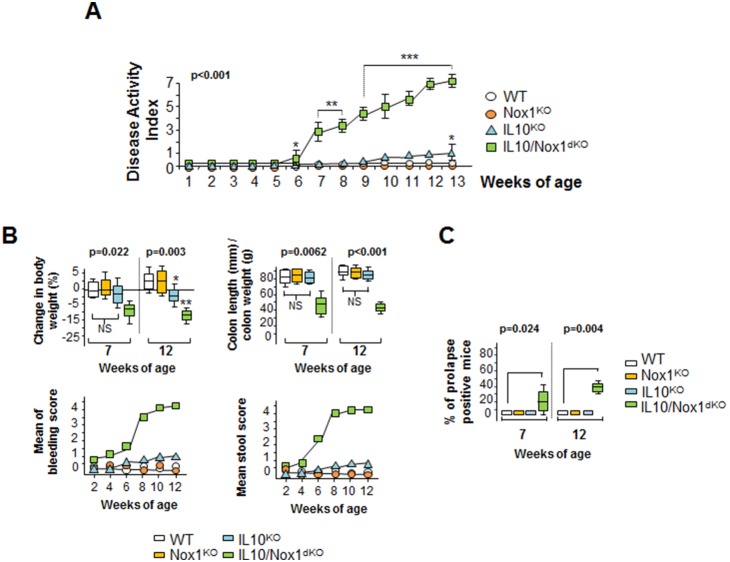
Spontaneous colitis in IL10/Nox1^dKO^ mice. (**A**) The DAI is measured daily in WT (n = 10), Nox1^KO^ (n = 10), IL10^KO^ (n = 10), and IL10/Nox1^dKO^ (n = 25) mice. Statistics: *p*-values for Kruskal-Wallis non-parametric analysis are shown, Dunn’s multiple comparison test versus WT; *p<0.05, **p<0.01, ***p<0.001. (**B**) Clinical symptoms of IL10/Nox1^dKO^ mice. Body weight changes, rectal bleeding and stool scores were assessed daily. The weight-to-length ratio was determined for each individual colon of WT (n = 10), Nox1^KO^ (n = 10), IL10^KO^ (n = 10), and IL10/Nox1^dKO^ (n = 25) mice aged 7 and 12 weeks. Statistics: box plots show median, quartiles, and range; *p*-values for Kruskal-Wallis non-parametric analysis are shown, Dunn's multiple comparison test *vs*. WT, NS, not significant. (**C**) Percentage of WT (n = 10), Nox1^KO^ (n = 10), IL10^KO^ (n = 20), and IL10/Nox1^dKO^ (n = 30) mice with prolapse at 7 and 12 weeks of age. Statistics are as in (B).

**Figure 2:**
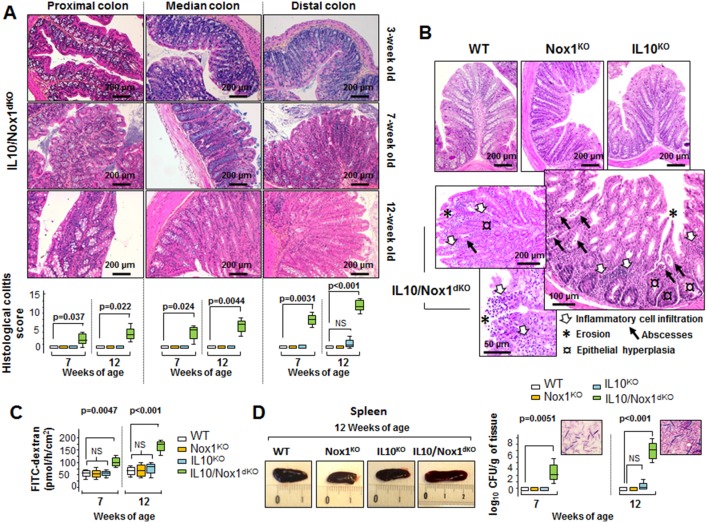
Clinical and histological features of IL10/Nox1^dKO^ mice. (**A**) Upper panel- Representative histological H&E staining of sections of the proximal, median, and distal colons of IL10/Nox1^dKO^ mice aged 3, 7, and 12 weeks. Lower panel- Histological colitis scores were determined at 7 and 12 weeks of age from proximal, median, and distal colon sections (n = 15/genotype). Statistics: box plots show median, quartiles, and range; *p*-values for Kruskal-Wallis non-parametric analysis are shown, Dunn's multiple comparison test *vs*. WT, NS, not significant. (**B**) Representative histology of normal distal colon of 12-week old WT, Nox1^KO^, IL10^KO^ mice and examples of inflammation in the distal colon of 12-week old IL10/Nox1^dKO^ mice. (**C**) Permeability of FITC-dextran in three different segments of the distal colon of WT, Nox1^KO^, IL10^KO^, and IL10/Nox1^dKO^ mice (n = 5/group) aged 7 and 12 weeks incubated in Ussing chambers. Statistics are as in (A). (**D**) Left panel - Representative image of the spleen of 12-week old IL10/Nox1^dKO^ mice *vs*. WT and single KO mice (scale in cm). Right panel - Quantification of viable bacteria translocated to the spleen of WT, Nox1^KO^, IL10^KO^, and IL10/Nox1^dKO^ mice (n = 5/group) aged 7 and 12 weeks. Results are presented as log_10_ CFU/g of tissue. Inserts show the presence of bacteria in the spleen. Identification of bacteria by 16SrRNA revealed mainly the presence of endogenous gut bacteria. Statistics are as in (A).

IL10/Nox1^dKO^ mice (n = 150) spontaneously developed clinical signs of colitis from 6–7 weeks of age and disease activity index (DAI) scores worsened with age (Fig. 1). Although some IL10^KO^ mice showed a slight weight loss at 13 weeks of age (Fig. 1B), none of them developed enterocolitis during the period studied. Classical signs of colitis including swollen, distended colon with bleeding and enlarged mesenteric lymph nodes were present. The histopathological analysis showed that in 3-week old IL10/Nox1^dKO^ mice, no histological signs of inflammation were present in the entire colon (Fig. 2A). Inflammation started at 6–7 weeks of age with typical proximal progression from the rectum to involvement of most or all the colon at 12 weeks of age (Fig. 2A) with no signs of ileitis (data not shown). Histologically, colitis features were similar to those observed in human UC, *i.e*. polymorphonuclear infiltrates, crypt abscesses, edema, focal epithelial erosion, crypt loss, and frank ulcerations without granulomas (Fig. 2B).

Consistently with the severity of colitis and epithelial damage, 7- and 12-week old IL10/Nox1^dKO^ mice had a barrier dysfunction characterized by an increased colonic permeability measured with FITC-dextran which worsened with age (Fig. 2C). Moreover, these mice had splenomegaly which was correlated with an increased commensal Gram-negative bacteria translocation which increased with colitis progression (Fig. 2D). As expected, no bacteria were found in the spleen of WT, Nox1^KO^, or IL10^KO^ mice (Fig. 2D).

### Immunopathology in IL10/Nox1^dKO^ mice

To study the immune response profile in our model, colon samples of 12-week old mice were collected and various cytokines were analyzed at both the mRNA and protein levels in all genotypes (Fig. S1A–B). IL1-β, TNFα, IL13, IL6, IL17A, and IFNβ expression levels were significantly increased in the colon of IL10/Nox1^dKO^ mice compared to other genotypes, particularly in the distal colon.

A significant increase in the percentage of CD4^+^ T cells including CD4^+^ T cell effectors and FoxP3^+^ T_reg_ cells and a decrease in the percentage of CD8^+^ T cells were detected in the lamina propria of IL10/Nox1^dKO^ mice compared to WT (Fig. S2). Furthermore, there was a trend toward an increase in CD11c^+^ dendritic cells in the colonic lamina propria of IL10/Nox1^dKO^ mice suggesting the contribution of both innate and adaptive immunity in this model. A massive infiltration of CD3^+^ lymphocytes (Fig. S3A) including CD4^+^ CD25^+^ FoxP3^+^ T_reg_ cells (Fig. S3B) was observed in the inflamed colon and to a lesser extent in the spleen only in IL10/Nox1^dKO^ mice (Fig. S3C) as previously reported in UC [29].

To determine whether the genotype of hematopoietic lineages affected colitis, we generated bone marrow chimeric mice for which recipients and donors were WT (CD45.1) and WT, IL10^KO^, and IL10/Nox1^dKO^ mice (CD45.2), respectively. Mice were studied 16 weeks after transplantation and a full chimerization assessed through surface staining of bone marrow cells was observed (Fig. S3D). The disease did not develop in irradiated WT mice with IL10^KO^ or IL10/Nox1^dKO^ bone marrow showing that the colitis could be mainly inherent to epithelial cells rather than hematopoietic lineages in IL10/Nox1^dKO^ mice (Fig. S3E). However, it is noteworthy that the reconstitution of IL10/Nox1^dKO^ mice with bone marrow from WT donors could be biased since histological signs of colitis were already present before irradiation and bone marrow transplantation. Unfortunately, this major bias, related to the early onset of colitis in IL10/Nox1^dKO^ mice, makes the reverse chimera uninformative.

### IL10/Nox1^dKO^ mice develop colitis-associated colonic dysplasia and cancer

UC is associated with a higher risk of occurrence of dysplasia and colorectal cancer [30]. We investigated whether IL10/Nox1^dKO^ mice had longstanding colonic disease complications by analyzing the late colonic evolution in 8-month old IL10/Nox1^dKO^ mice (n = 35). 10 mice developed dysplasia (30%), 14 mice developed colonic cancer (40%), and 5 had multifocal dysplasia and cancer (15%) (Fig. S4).

### IL10/Nox1^dKO^ mice exhibit early goblet cell alteration before severe colitis and signs of regenerative and apoptotic responses

**Figure 3:**
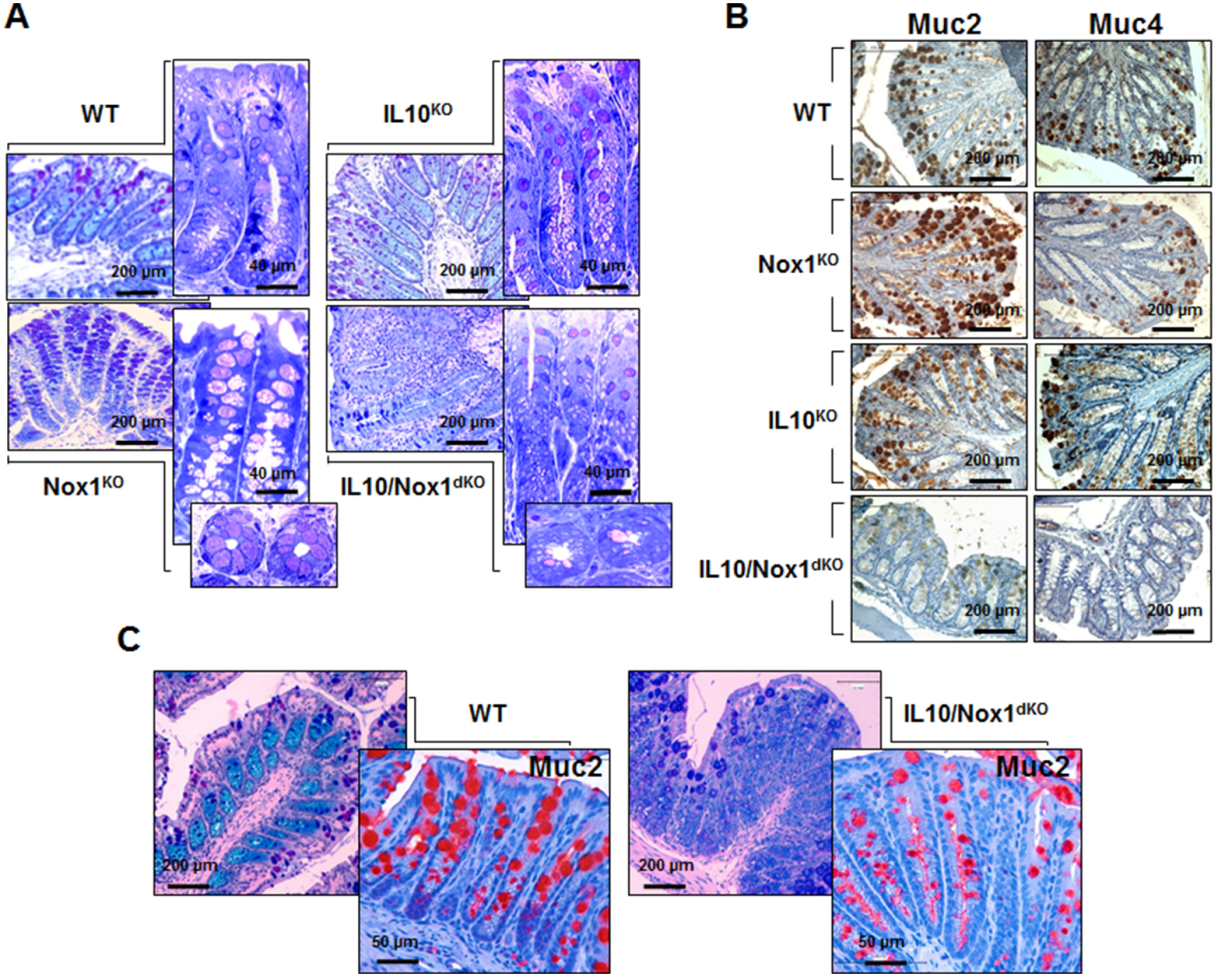
Altered goblet cells and mucin expression in IL10/Nox1^dKO^ mice. (**A**) Representative sections of the distal colon of 7-week old WT (n = 10), Nox1^KO^ (n = 10), IL10^KO^ (n = 10), and IL10/Nox1^dKO^ (n = 20) mice stained with AB/PAS. The distal colon of 7-week old WT (n = 5), Nox1^KO^ (n = 5), IL10^KO^ (n = 5), and IL10/Nox1^dKO^ (n = 5) mice is shown both in transverse and longitudinal semi-thin sections. (**B**) Immunohistological analysis of Muc2 and Muc4 in distal colonic sections of 7-weeks old WT (n = 5), Nox1^KO^ (n = 5), IL10^KO^ (n = 5), and IL10/Nox1^dKO^ (n = 5) mice. (**C**) Representative sections of the distal colon of 3-week old WT (n = 5) and IL10/Nox1^dKO^ (n = 5) mice stained with AB/PAS (upper panels) or with anti-Muc2 antibody (lower panels).

**Figure 4:**
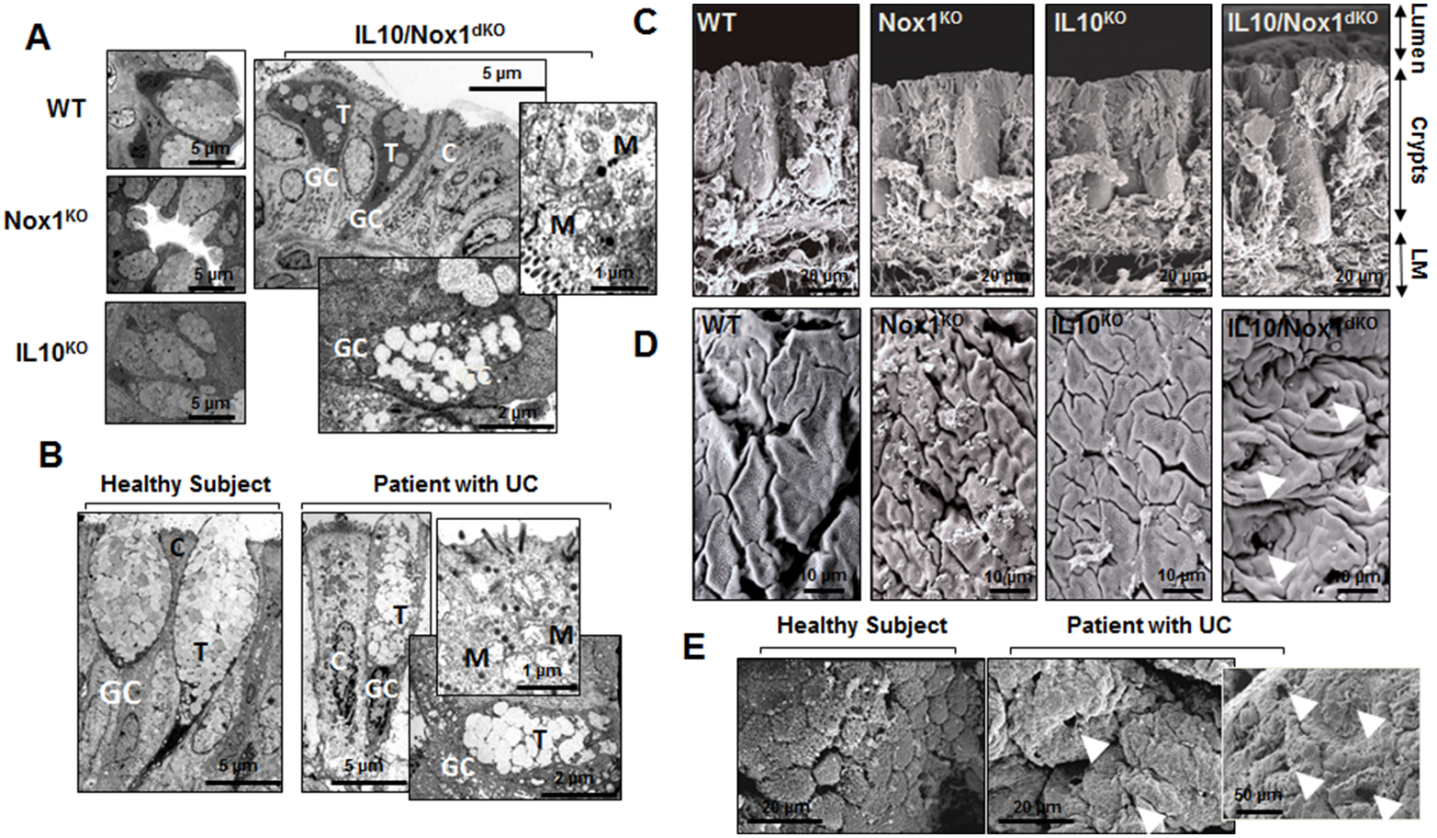
Similar ultrastructural abnormalities in the colonic epithelium of IL10/Nox1^dKO^ mice and patients with UC. (**A**) Representative transmission electron micrographs of the unaffected colon of 4-week old WT (n = 5), Nox1^KO^ (n = 5), IL10^KO^ (n = 10), and IL10/Nox1^dKO^ (n = 10) mice. (**B**) Representative transmission electron micrographs of the unaffected colon of 10 healthy subjects and 10 patients with UC. Note that IL10/Nox1^dKO^ mice display morphological goblet cell abnormalities similar to those found in patients with UC including reduced size of thecae containing stored mucins, immature mucus granules, and swollen mitochondria. (**C**) Representative scanning electron micrographs (SEM) of distal colonic crypts of 6-week old WT (n = 5), Nox1^KO^ (n = 5), IL10^KO^ (n = 10), and IL10/Nox1^dKO^ (n = 10) mice. Note that the Lieberkhün’s crypts are longer in IL10/Nox1^dKO^ mice. (**D**) Representative scanning electron micrographs of the distal colonic epithelium of 6-week old WT (n = 5), Nox1^KO^ (n = 5), IL10^KO^ (n = 8), and IL10/Nox1^dKO^ (n = 10) mice. Note the inappropriate pattern of crypt openings (arrowheads) on SEM with enlarged extrusive zones and dilated gland lumen in the distal colon of IL10/Nox1^dKO^ mice. (**E**) Representative SEM of the unaffected colonic mucosa of 10 healthy subjects and 10 patients with UC. Note the regular pattern of crypt openings with diffuse edema, enlarged extrusive zones, and dilated gland lumen similar to those found in IL10/Nox1^dKO^ mice. Abbreviations: GC: goblet cell, C: colonocyte, T: thecae, LM: lamina propria, M: mitochondria.

Mucins were rare in the colonic epithelium of 6–7-week old IL10/Nox1^dKO^ mice associated with a loss of goblet cells in ulcerated sites (Fig. 3A). Accordingly, Muc2 and Muc4 protein levels were reduced in inflamed colonic areas of IL10/Nox1^dKO^ mice (Fig. 3B). Reduced mature goblet cell number and size and decreased Muc2 expression were detected early in the distal colon of 3–4-week old IL10/Nox1^dKO^ mice when no inflammation was detected, suggesting that the defect in goblet cells could precede histologically detectable inflammation (Fig. 3C). Aberrant goblet cells with a few immature thecae associated with reduced mucus were observed in the colon of IL10/Nox1^dKO^ mice and UC patients (Fig. 4A–B).

The number of PCNA- and phospho-histone 3-positive cells was increased in the colonic sections of IL10/Nox1^dKO^ mice suggesting an increased epithelial proliferation (Fig. S5). A ∼30% increase in crypt length was found in IL10/Nox1^dKO^ mice using scanning electron microscopy (SEM) (Fig. 4C and Fig. S6A). Interestingly, numerous identical ultrastructural alterations were found in the colonic mucosa of IL10/Nox1^dKO^ mice and UC patients on SEM (Fig. 4D–E). Despite the increased colonic proliferation, the staining and quantitative assessment of active caspase 3-positive apoptotic cells in the villous epithelium of IL10/Nox1^dKO^ mice suggested that the decreased number of goblet cells was mainly due to an increased apoptosis in the colon (Fig. S6B–E).

### IL10/Nox1^dKO^ mice exhibit impaired ER stress response in epithelial cells

To assess the role of goblet cells in ER stress-induced colitis, WT and goblet cell-overexpressing Nox1^KO^ mice received orally dextran-sodium-sulfate (DSS) (Fig. S7) or rectally 2,4,6-trinitrobenzene sulfonic acid (TNBS) (Fig. S8). There was no significant difference in DAI scores or in the histological damage of the colonic mucosa between the two mouse models. This demonstrates that chemically-induced inflammation is probably independent of the increase in goblet cells. It should be noted that no difference in clinical and histological scores was observed between WT and Nox1^KO^ mice fed with different doses of DSS varying from 2% to 5% (data not shown). On the other hand, the acute ER stress induced by tunicamycin (TM) treatment, a canonical ER stress inducer, induced colitis which resulted in a decreased goblet cell number, inflammatory infiltrate, and erosion of the colonic epithelium in both WT and Nox1^KO^ mice, and was exacerbated in Nox1^KO^ mice (Fig. S9). These data suggest that goblet cells could directly participate to the development of ER stress-induced colitis.

**Figure 5:**
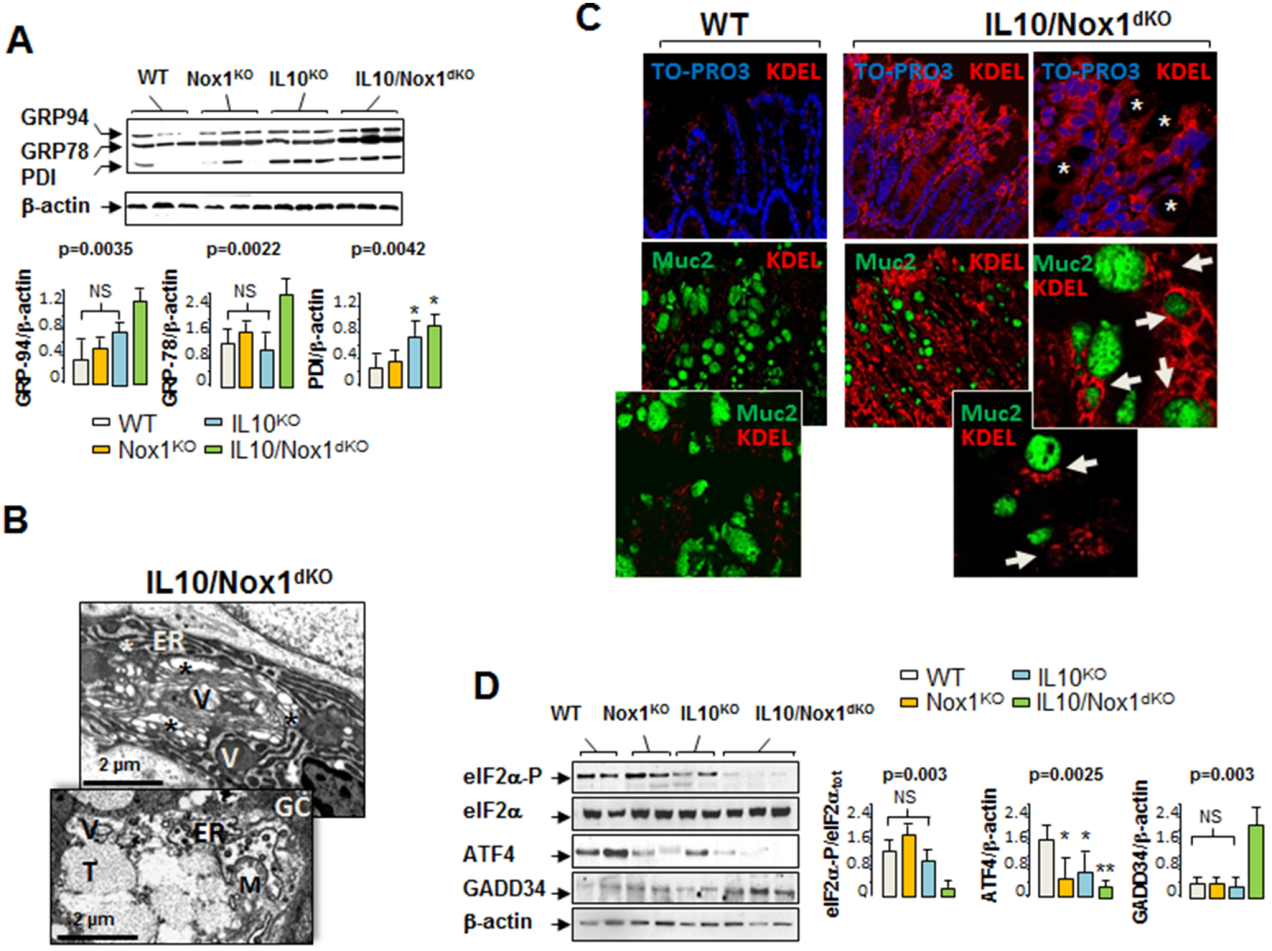
Evidence of altered UPR in IL10/Nox1^dKO^ mice. (**A**) Representative immunoblot analysis of chaperone expression in the distal colon of 3–4-week old WT, Nox1^KO^, IL10^KO^, and IL10/Nox1^dKO^ mice (n = 9/group) using an anti-KDEL antibody. β-actin was used as loading control and densitometric analyses are shown. *P*-values for Kruskal-Wallis non-parametric analysis are shown, Dunn’s multiple comparison test versus WT, NS; not significant. (**B**) Ultrastructural evidence of ER stress in the colonic epithelium of 4-week old IL10/Nox1^dKO^ mice (n = 10). Representative transmission electron micrographs of goblet cells showing dilation of the endoplasmic reticulum (asterisks). Abbreviations: GC, goblet cell, ER, endoplasmic reticulum, T, thecae, M, mitochondria, V, vacuoles. (**C**) Confocal microscopy of colonic sections of WT and IL10/Nox1^dKO^ mice stained with antibody against KDEL sequence (red) (upper panel). Original magnification (x40). The panel on the right-hand side represents a higher magnification (x60). Goblet cell thecae are identified by white asterisks. Nuclei (blue) are stained with TO-PRO-3 iodide. Lower panels: double indirect immunofluorescence of colonic sections of WT and IL10/Nox1^dKO^ mice stained with antibodies against Muc2 (green) and KDEL sequence (red). Original magnification (x40). Photomicrographs are representative sections of five mice for each genotype. Inset boxes are enlarged views showing co-expression of both markers in goblet cells (white arrows). Original magnification (x60). (**D**) Representative immunoblot analysis of indicated protein expression in the distal colon of 3–4-week old WT, Nox1^KO^, IL10^KO^, and IL10/Nox1^dKO^ mice (n = 9/group). β-actin served as a loading control. The P-eIF2β/eIF2β ratio was measured and densitometric analyses are shown. Statistics are as in (A*)*.

As previously described in the unaffected mucosa of UC patients [5], 3–4-week old IL10/Nox1^dKO^ mice exhibited chronic ER stress alterations in the colonic mucosa prior to severe colitis. IRE1 and ATF6α UPR branches were activated in colonic epithelial cells as shown by the increased *XBP-1* mRNA splicing, the induction of major ER chaperones such as GRP78/Bip, GRP94, and PDI at both the mRNA and protein levels, and the dilated cisternae and gross distortion of the ER in goblet cells (Fig. 5A–B, and Fig. S10A). Epithelial cells with increased signal intensity for ATF6α and GRP78/Bip were found in the upper villus regions of the colon of IL10/Nox1^dKO^ mice (Fig. S10B). The expression of KDEL-containing proteins (motif of permanent ER retention common to ER stress-induced chaperones) was strongly increased in the colonic epithelium of IL10/Nox1^dKO^ mice compared with WT mice (Fig. 5C). Additionally, ER resident KDEL-containing chaperones were co-expressed in Muc2-positive cells suggesting the presence of unabated ER stress in goblet cells of IL10/Nox1^dKO^ mice (Fig. 5C).

We next investigated the efficiency of the integrated stress response mediated by the PERK/eIF2α/ATF4 pathway in the colonic mucosa of IL10/Nox1^dKO^ mice. Note that the defective eIF2α phosphorylation correlating with low ATF4 mRNA and protein expression was observed in the colonic mucosa of both IL10/Nox1^dKO^ mice (Fig. 5D and Fig. S10A) and patients with inactive UC [5]. As we have previously shown in humans [5], the increased expression of PPP1R15A/GADD34, a stress-inducible protein which recruits the catalytic subunit of the protein phosphatase 1 (PP1c) to promote eIF2α dephosphorylation, was associated with a reduced eIF2α phosphorylation (Fig. 5D).

### Nox1 and IL10 differentially regulate the ER stress in colonic goblet cells

**Figure 6:**
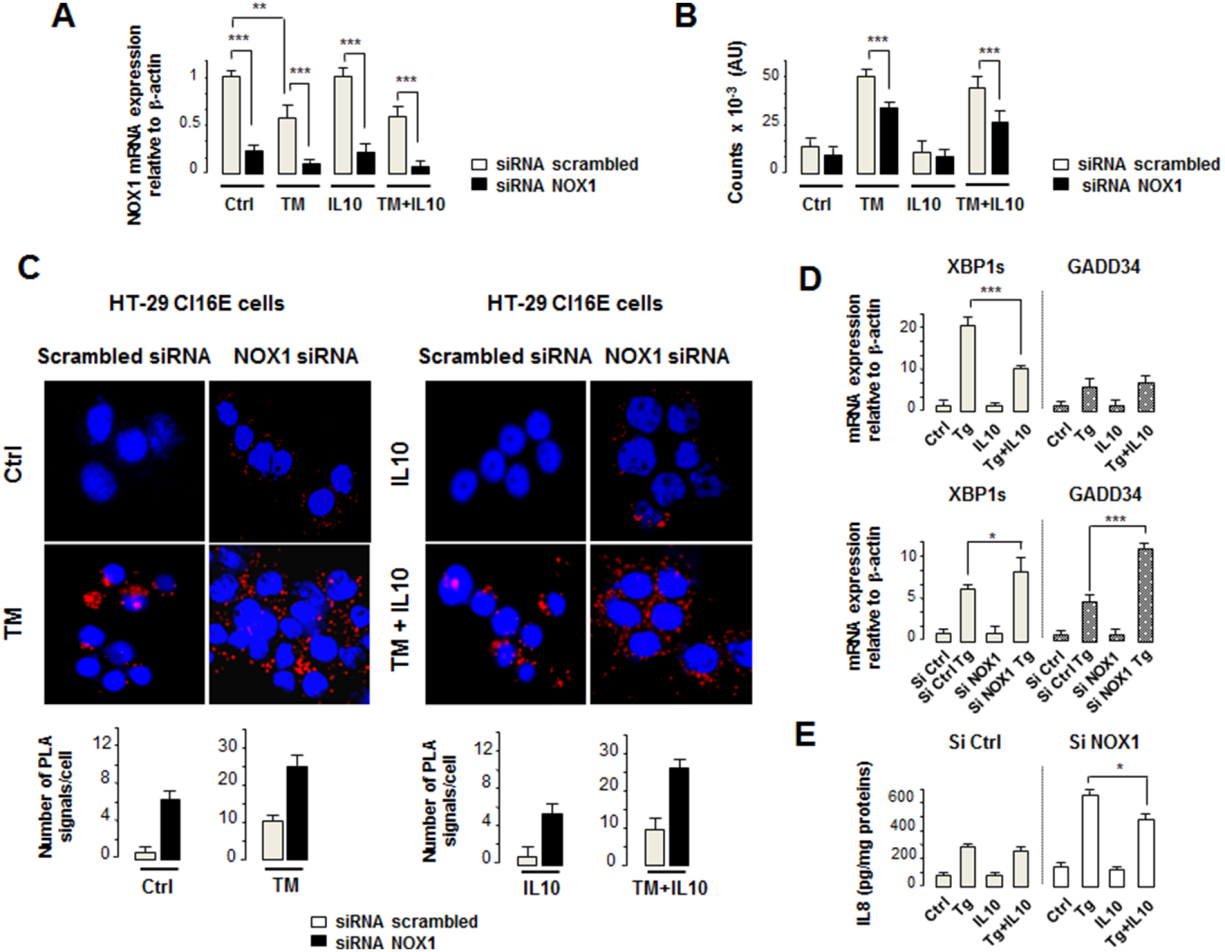
Nox1 and IL10 negatively regulate the UPR in cultured intestinal goblet cells. (**A**) HT-29Cl16E cells carrying siRNA scrambled or Nox1 siRNA were treated in triplicate with vehicle (Ctrl), IL10 (50 ng/ml), TM (5 µg/ml), or IL10+TM. *NOX1* mRNA levels were determined by qPCR and normalized to β-actin with the mean ratio of the control group corrected to 1. Statistics: *p*-values for Kruskal-Wallis non-parametric analysis are shown, Dunn’s multiple comparison test versus Ctrl, **p<0.01, ***p<0.001. (**B**) eIF2α phosphorylation was measured using an Alphascreen *SureFire* P-Ser^51^-eIF2α assay in three independent experiments (mean +/− SD). (**C**) Upper panel – HT-29Cl16E cells carrying siRNA scrambled or Nox1 siRNA were treated with vehicle (Ctrl), IL10 (50 ng/ml), TM (5 µg/ml), or IL10+TM. Proximity between PP1c and GADD34 was detected by PLA. Nuclei were stained with TO-PRO-3 iodide. Confocal photomicrographs are representative of four independent experiments (original magnification x40). Lower panel – Quantification of PLA signals for PP1c and GADD34 proximity (n = 8 per condition). Fluorescent signals were counted using Imaris software and the average number of spots per cell is represented (mean ± SD). (**D**) Upper panel - HT-29Cl16E cells were treated in triplicate with vehicle (Ctrl), thapsigargin (Tg, 5 µM), IL10 (50 ng/ml), or IL10+Tg. Lower panel - HT-29Cl16E cells carrying scrambled (si) or Nox1 siRNA (si NOX1) were treated in triplicate with vehicle (Ctrl) or Tg (5 µM). *XBP1s* and *GADD34* mRNA levels were determined by qPCR and normalized to β-actin Statistics: *p*-values for Kruskal-Wallis non-parametric analysis are shown, Dunn’s multiple comparison test versus Ctrl, *p<0.05, ***p<0.001. (**E**) Concentration of IL8 in supernatants from HT-29Cl16E cells carrying scrambled (si) or Nox1 siRNA (si NOX1) treated in triplicate with vehicle (Ctrl),), thapsigargin (Tg, 5 µM), IL10 (50 ng/ml), or IL10+Tg (mean S.D).

To further investigate the mechanism by which IL10 and Nox1 regulated the ER stress and triggered inflammation in goblet cells, an *in vitro* model of intestinal mucus-secreting cells, the human HT-29Cl16E cells, was used [31]. The ability of Nox1 and IL10 to elicit or repress eIF2α phosphorylation by modulating the formation of PP1c/GADD34 complexes under stress conditions was first assessed. To this end, HT-29Cl16E cells carrying scrambled or Nox1 siRNAs were treated with TM in the presence or absence of IL10. *Nox1* mRNA level was reduced by >75–80% in cells transfected with Nox1 siRNA compared to control cells (Fig. 6A). TM significantly reduced *Nox1* mRNA level in the two cell populations (Fig. 6A). EIF2α phosphorylation and the formation of PP1c/GADD34 complexes were next analyzed using the Alphascreen SureFire P-Ser^51^-eIF2α assay and Duolink proximity ligation assay, respectively. As expected, TM induced eIF2α phosphorylation which was balanced by an increased number of PP1c/GADD34 complexes in controls cells carrying scrambled siRNA (Fig. 6B–C). No significant changes were observed in eIF2α phosphorylation and the formation of PP1c/GADD34 complexes in control cells treated with IL10 alone or in combination with TM (Fig. 6A–B) excluding a potential regulatory role of IL10 on the integrated stress response. In contrast, TM-induced eIF2α phosphorylation was significantly reduced in cells carrying Nox1 siRNAs associated with a >5-fold increase in the number of PP1c/GADD34 complexes regardless of the presence of IL10 (Fig. 6B–C). Similar imbalance was observed with Thapsigagin (Tg), another ER stressor which inhibits the calcium pump SERCA (data not shown). It is noteworthy that Nox1 silencing slightly increased the number of PP1c/GADD34 complexes in the absence of TM compared to control cells (Fig. 6C). These data provide evidence that Nox1 silencing could induce eIF2α dephosphorylation in stressed goblet cells by enhancing PP1c/GADD34 interactions. Interestingly, the reduced eIF2α phosphorylation was also observed in the distal colonic mucosa of TM-treated Nox1^KO^ mice (Fig. S10 C) suggesting that changes in Nox1 expression impaired the TM-induced integrated stress response associated with an increased susceptibility to inflammation (see Fig. S9). Although IL10 did not exert any regulatory effect on the integrated stress response in our experimental conditions, it reduced IRE1-dependent *XBP1* splicing induced by Tg (Fig. 6D) in accordance with the suppressive effect of IL10 on the ER stress via the IL10R/Stat1–3 signaling pathway previously proposed by Hasnain *et al*. [2]. Consistently with the absence of suppressive effect of IL10 on eIF2α phosphorylation, no change in Tg-induced *GADD34* mRNA expression was observed upon IL10 treatment (Fig. 6D). Of note, IL10 did not modify Tg- or TM-induced *ATF4* and *CHOP* mRNA levels (data not shown). In contrast, Nox1 silencing strongly increased the ER stress-induced *GADD34* mRNA and to a lesser extent *XBP1* splicing, suggesting a crucial role of Nox1 in the regulation of the integrated stress response and more broadly of the UPR (Fig. 6D). Furthermore, Nox1 deficiency induced an increased secretion of the pro-inflammatory chemokine IL8 by goblet cells in the presence of Tg (Fig. 6E). IL10 slightly but significantly reduced the Tg-induced secretion of IL8 by cells carrying Nox1 siRNA (Fig. 6E) suggesting that the deregulated ER stress in goblet cells could initiate the inflammation in the colonic mucosa.

Altogether, these data demonstrated the multifaceted role of IL10 and Nox1 in the regulation of the ER stress in goblet cells, improving the course of UPR, suppressing proinflammatory signaling originating from goblet cells, and facilitating the mucosal barrier function.

### Salubrinal treatment prevents colitis in IL10/Nox1^dKO^ mice

**Figure 7:**
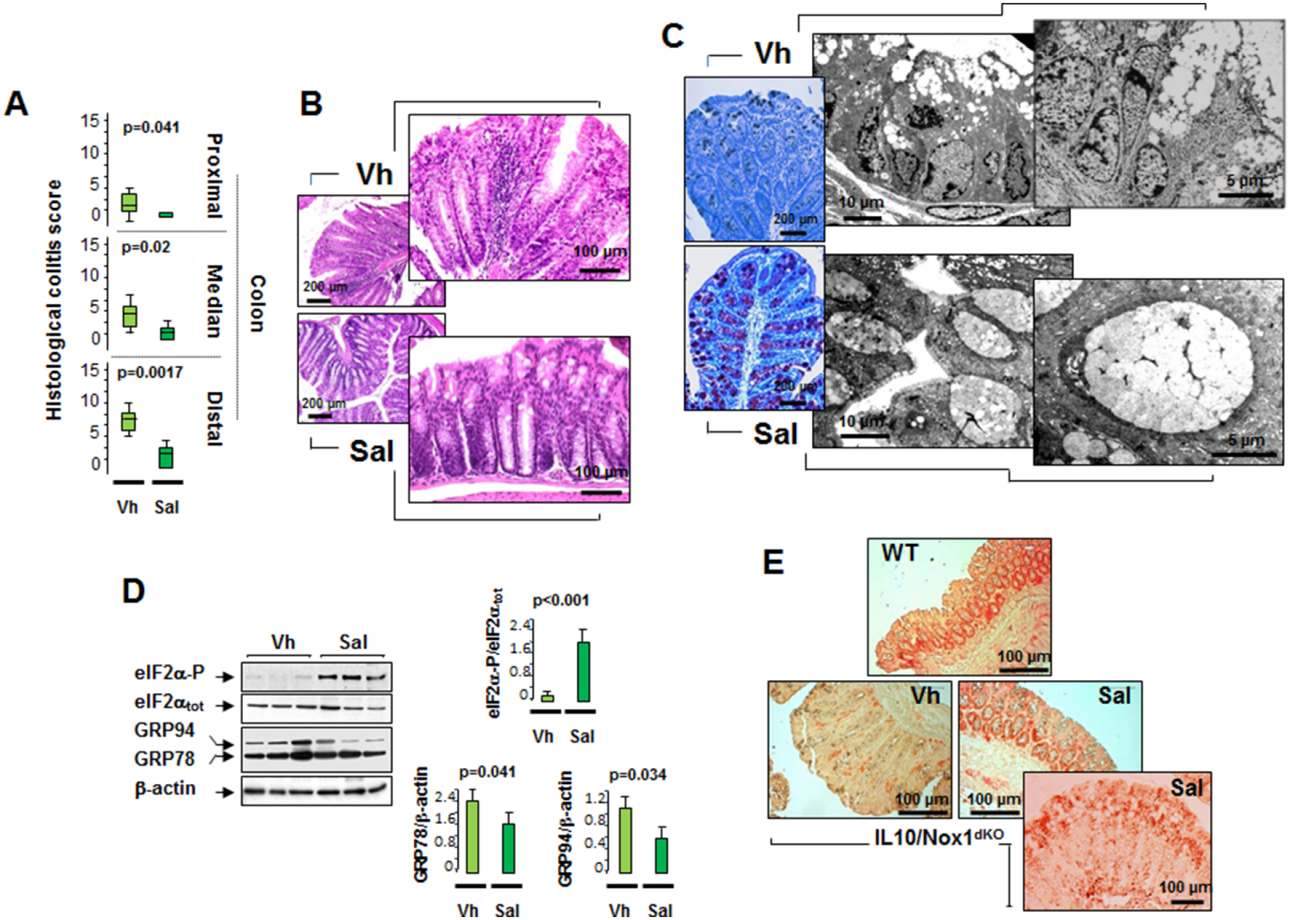
Salubrinal rebalances the altered ER stress and prevents colitis. Three-4-week old IL10/Nox1^dKO^ mice received 1 mg/kg salubrinal (Sal) intraperitonally or vehicle (Vh) 3 times per week for 3 weeks. (**A**) Histological colitis scores were determined at 6–7 weeks of age from H&E-stained colonic sections. Statistics: box plots show median, quartiles, and range; Mann-Whitney U-test, *p*-values are shown. (**B**) Representative H&E-stained sections of the distal colon of Vh- (n = 10) or Sal (n = 15)-treated IL10/Nox1^dKO^ mice. (**C**) Left panels - Goblet cell staining with blue alcian/periodic acid Schiff stain on distal colonic sections of Vh- or Sal-treated IL10/Nox1^dKO^ mice (n = 15/group). Right panels - Representative transmission electron micrographs of the distal colon of Vh- (n = 6) or Sal- (n = 8) treated IL10/Nox1^dKO^ mice. (**D**). Representative immunoblot analysis of indicated protein expression in the distal colon of Vh- (n = 10) or Sal- (n = 15) treated IL10/Nox1^dKO^ mice aged 6–7 weeks. β-actin is used as loading control. The P-eIF2α/eIF2α ratio was measured and densitometric analyses are shown. *P*-values for Mann-Whitney U-test analysis are shown. (**E**) Representative immunohistological analysis of P-eIF2αβ (Ser^51^) in WT (n = 8), Vh- (n = 10) or Sal- (n = 15) treated IL10/Nox1^dKO^ mice aged 6–7 weeks. Note that the P-eIF2α epithelial staining in salubrinal-treated IL10/Nox1^dKO^ mice is similar to that of WT mice.

Our findings highlighted that the eIF2α phosphorylation was altered in both UC patients [5] and IL10/Nox1^dKO^ mice (this study) prior to colitis associated with an increased formation of the GADD34 and PP1c/GADD34 complex. To test whether a selective pharmacological inhibitor of PP1c/GADD34-mediated eIF2α dephosphorylation could prevent colitis, IL10/Nox1^dKO^ mice were treated with salubrinal [32] for three weeks. Salubrinal strongly reduced the histological colitis score throughout the colon, markedly prevented immune cell infiltration, and restored the intact mucosal architecture with normal goblet cells (Fig. 7A–C). Salubrinal sustained eIF2α phosphorylation and reduced GRP78/Bip and GRP94 expression in IL10/Nox1^dKO^ mice (Fig. 7D). Interestingly, we demonstrated that salubrinal-induced eIF2α phosphorylation was mainly detected in colonic epithelial cells (Fig. 7E). Finally, pro-inflammatory cytokines and percentage of colonic and splenic T_reg_ cells were decreased in salubrinal-treated IL10/Nox1^dKO^ mice (Fig. S11) highlighting a restoration of the colonic mucosal homeostasis.

### Altered IL10 and NOX1 expression in the non-inflamed colonic mucosa of UC patients

**Figure 8:**
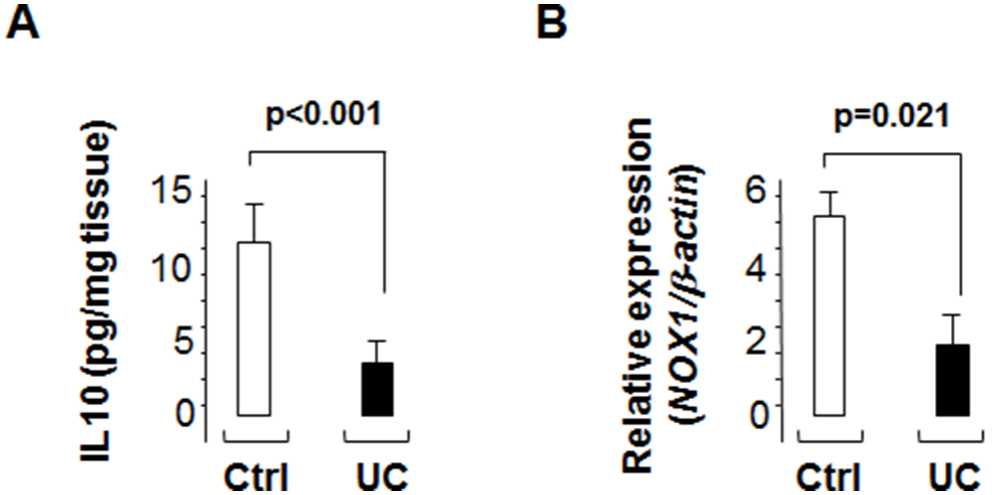
The IL10 protein and *NOX1* mRNA are detected in the non-inflamed colonic mucosa of controls (Ctrl, n = 12) and UC patients (n = 12) by ELISA and qPCR, respectively. Statistics: Mann-Whitney U-test (*p-*values shown).

As NOX1 is mainly expressed in colonic epithelial cells in humans [24], [33], [34], [35] we hypothesized that abnormal IL10 and NOX1 levels could be observed in UC patients. We thus assessed IL10 and NOX1 levels in the non-inflamed colonic mucosa of UC patients (n = 12) and healthy controls (n = 12) (Fig. 8). UC tissue biopsy specimens from non-inflamed regions were sampled during endoscopy. Unaffected areas were defined as mucosal regions containing about 85–90% of epithelial cells without any macroscopic, endoscopic, and histological signs of inflammation. Basal expressions of both IL-10 (*P*<0.001, protein level) and *NOX1* (p = 0.02, mRNA level) were significantly decreased in the non-inflamed colonic mucosa of UC patients compared to healthy subjects. These findings highlighted the clinical relevance of the IL10/Nox1^dKO^ mouse model for UC.

## Discussion

In this study, we showed that both immunological and epithelial deficiencies in mice lacking the anti-inflammatory cytokine IL10 and Nox1 sensitized the colon to spontaneously develop severe colitis and mimicked all clinical and histological characteristics of UC. Consistently, we showed that both IL10 and NOX1 expression levels were decreased in the non-inflamed colonic mucosa of UC patients compared to healthy controls. We can assume that IL10 and Nox1 could play a central role in UC pathogenesis and cooperate to modulate the UPR and inflammation, particularly in goblet cells. We identified an early defect of eIF2α phosphorylation associated with an increased GADD34 expression in the non-inflamed colon of IL10/Nox1^dKO^ mice as previously reported in UC patients [5]. We found that Nox1 invalidation in goblet cells increased both GADD34 transcription and the number of PP1c/GADD34 complexes responsible for eIF2α dephosphorylation under stress conditions. These data highlight for the first time that Nox1 is directly involved in the negative regulation of the integrated stress response. Consistently, Nox1^KO^ mice which exhibited a high number of goblet cells in the colon [20] developed severe colitis after acute treatment with TM and failed to induce eIF2α phosphorylation. Although we can assume that the products of Nox1, ROS, could be responsible for the preservation of eIF2 phosphorylation under prolonged ER stress through a negative control of the formation of PP1c/GADD34 complexes, the precise mechanism by which Nox1 inhibits the integrated stress response and controls inflammation remains to be clarified.

Interestingly, Nox1 invalidation in HT29Cl16E cells elicited Tg-induced IL8 release which was partially limited when cells were treated with IL10, suggesting that both IL10 and Nox1 are involved in the regulation of the ER stress-dependent inflammatory signaling in the epithelial barrier. A previous report supported that bacterial peptides, such as N-formyl peptide (fMLP), could interact with goblet cells and induce the release of chemokines including IL8, leading to neutrophil recruitment and mucus depletion [36]. In parallel, Nox1 seems to play a crucial role downstream of the fMLP receptor in intestinal epithelial cells by promoting the mucosal wound repair through ROS production [22], [37]. These findings support the hypothesis that early events in mucosal inflammation take place when both Nox1 and IL10 suppressive effects on the ER stress are deficient in goblet cells.

Furthermore, IL10 is known to modulate the ER stress response in intestinal epithelial cells. Characterizing the proteome of intestinal epithelial cells from *Enterococcus faecalis*-monoassociated IL10^KO^ mice revealed an inadequate response to oxidative and ER stresses associated with increased GRP78/Bip expression levels [9], [38]. Hasnain *et al.* [2] have demonstrated that IL10 deficiency combined with the *Winnie* missense misfolding mutation in Muc2 [3] exacerbated the ER stress in goblet cells and resulted in severe colitis. The authors reported that IL10 directly suppressed TM-induced *XBP1* splicing and maintained mucin production under stress condition through the up-regulation of genes involved in the mucin folding (Agr2) and ER-associated degradation process (ERAD) in goblet cells. Interestingly, our data showed that IL10 alleviated the ER stress through the inhibition of the IRE1/XBP1 pathway without affecting the eIF2 phosphorylation. IL10 had no significant effect on the TM-induced formation of PP1c/GADD34 complexes, suggesting that Nox1 and IL10 alleviate the ER stress by acting on distinct UPR branches. We can assume that IL10 and Nox1 synergize to regulate the ER stress in goblet cell functions and inflammatory process.

A growing body of evidence suggests that ER stress abnormalities in secretory cells could contribute to UC pathogenesis and therefore modulating the ER stress has therapeutic potential. In proof-of-concept experiments, we showed that salubrinal, which inhibits the PP1c/GADD34 activity, restored the eIF2α phosphorylation and prevented colitis in IL10/Nox1^dKO^ mice. These data suggest that future selective small molecules targeting eIF2α dephosphorylation could be novel strategies for UC.

The morphological, phenotypical, and functional alterations observed highlighted the relevance of our murine model for studying multiple aspects of UC including its associated complications such as colitis-associated cancer. It is noteworthy that the C57/Bl6 genetic background is known to be resistant to neoplasia, and that only a few IL10 mice develop cancer as previously reported by Kanneganti et al. [39]. The IL10/Nox1^dKO^ model will help testing the preventive and curative effect, the long-term efficacy in maintaining remission, and probably the long-term properties in preventing cancer of current and future molecules. This is priceless because so far long-term comparative clinical studies testing colorectal cancer chemoprevention in UC patients are not feasible.

## Supporting Information

Figure S1 **Cytokine expression and leukocyte composition in IL10/Nox1^dKO^ mice.** (**A**) Quantitative reverse transcriptase-PCR array was performed on the distal colonic sections of 7-week old WT (n = 5), Nox1^KO^ (n = 5), IL10^KO^ (n = 5), and IL10/Nox1^dKO^ (n = 5) mice. Cytokine mRNA levels were normalized to GAPDH and expressed as relative fold change to the mean expression in WT mice. *P*-values for Kruskal-Wallis non-parametric analysis are shown; Dunn’s multiple comparison test vs. WT, NS, not significant. (**B**) Concentrations of different cytokines in supernatants from colonic lysates of 12-week old WT (n = 7), Nox1^KO^ (n = 8), IL10^KO^ (n = 8), and IL10/Nox1^dKO^ (n = 10) mice. Statistics: box plots show median, quartiles, and range; *p*-values for Kruskal-Wallis non-parametric analysis are shown, Dunn's multiple comparison test *vs*. WT, NS, not significant.
(TIF)

Figure S2 **Altered lamina propria leukocyte composition in IL10/Nox1^dKO^ mice.** Lamina propria mononuclear cells from the colon of WT (n = 5) and IL10/Nox1^dKO^ (n = 5) mice were stained for indicated markers and analyzed by flow cytometry, and expressed as a proportion of cells from the total live gate. Statistics: *p*-values for Mann-Whitney *U-test* are shown, NS; not significant.
(TIF)

Figure S3 **(A) Representative immunohistological analysis of CD3^+^ cells from distal colonic sections of 7-week old WT, Nox1^KO^, IL10^KO^, and IL10/Nox1^dKO^ mice (n = 5/group).** (**B**) Representative immunohistological analysis of Foxp3^+^ cells from distal colonic sections of 7-week old WT, Nox1^KO^, IL10^KO^, and IL10/Nox1^dKO^ mice (n = 5/group). (**C**) T_reg_ (CD4^+^ CD25^+^ Foxp3^+^) cell count in 7-week old IL10/Nox1^dKO^ mice expressed as a total lymphocyte percentage in the spleen. Statistics: box plots show median, quartiles, and range; *p*-values for Kruskal-Wallis non-parametric analysis are shown, Dunn's multiple comparison test *vs*. WT, NS, not significant. (**D**) Bone marrow stem cells were isolated from WT, IL10^KO^ or IL10/Nox1^dKO^ CD45.2/Ly5.2 mice (4–6-week old) and injected intravenously into WT CD45.1/Ly5.1 lethally-irradiated recipients. Mice were studied 16 weeks after transplantation and the chimerism was assessed by flow cytometry using the Ly5.1 and Ly5.2 markers. Mononuclear cells were stained for CD45, CD3, CD19 or CD11c then analyzed by flow cytometry (individual points are shown). (**E**) Representative H&E- (left panels) and AB/PAS (right panels)-stained distal colonic sections of recipient WT mice reconstituted with WT, IL10^KO^ or IL10/Nox1^dKO^ bone marrow show normal colonic morphology and goblet cells.
(TIF)

Figure S4 **Natural history of spontaneous colitis-associated cancer in 8-month old IL10/Nox1^dKO^ mice.** (**A**) Histopathological whole-mount view of Swiss-roll showing dysplasia and multifocal cancer lesions developed in the colon (**B**) Histopathological image of dysplasia-associated lesion or mass (DALM). (**C**) Ulceration and basal plasmocytosis. (**D**) Crypt abscesses and plasmocytosis. (**E**) Low-grade dysplasia. (**F**) High-grade dysplasia. (**G**) Invasive adenocarcinoma occurring in the submucosa.
(TIF)

Figure S5 **Colonic crypt proliferation is increased in IL10/Nox1^dKO^ mice.** Immunohistochemical analysis of the distal colonic sections of 7-week old WT (n = 5), Nox1^KO^ (n = 5), IL10^KO^ (n = 5), and IL10/Nox1^dKO^ (n = 5) mice stained with antibodies against the proliferating antigens (**A**) PCNA and (**B**) phospho-histone-3 (PH-3). The number of PCNA+ and PH3+ nuclei was counted in 10 and 50 consecutive crypts from proximal, median, and distal colon, respectively. Statistics: box plots show median, quartiles, and range; *p*-values for Kruskal-Wallis non-parametric analysis are shown, Dunn's multiple comparison test vs. WT, *p<0.05, NS, not significant.
(TIF)

Figure S6 **Increased proliferation and apoptosis in the colonic crypts of IL10/Nox1^dKO^ mice.** (**A**) Length of proximal, median, and distal colonic crypts in 6–7-week old WT (n = 10), Nox1^KO^ (n = 10), IL10^KO^ (n = 15), and IL10/Nox1^dKO^ (n = 15) mice. Statistics: box plots show median, quartiles, and range; *p*-values for Kruskal-Wallis non-parametric analysis are shown, Dunn's multiple comparison test *vs.* WT, NS, not significant. (**B**) The number of active caspase 3 positive cells was counted in 10 consecutive crypts from proximal, median, and distal colon of 7-week old WT (n = 10), Nox1^KO^ (n = 10), IL10^KO^ (n = 15), and IL10/Nox1^dKO^ (n = 15) mice. Statistics: box plots show median, quartiles, and range; *p*-values for Kruskal-Wallis non-parametric analysis are shown, Dunn's multiple comparison test *vs.* WT, NS, not significant. (**C**) Transmission electron micrographs of the distal colon of 7-week old IL10/Nox1^dKO^ mice (n = 5) reveal reduced size of goblet cell (GC) thecae (T), pycnotic GC nuclei with irregular edge (white arrows), altered mitochondria (M) and cytoplasm vacuolization, apoptotic fragments and vacuole containing condensed GC debris (black arrows). (**D**) Confocal microscopy of colonic sections of WT and IL10/Nox1^dKO^ mice stained with antibody against active caspase 3 (red). Original magnification (x40). (**E**) Representative immunohistological analysis of active caspase 3 in distal colonic sections of 6-week old WT (n = 5), Nox1^KO^ (n = 5), IL10^KO^ (n = 10), and IL10/Nox1^dKO^ (n = 10) mice. Magnification of the micrographs shows increased immunostaining of active caspase 3 in IL10/Nox1^dKO^ mouse GC.
(TIF)

Figure S7 **Susceptibility of WT and Nox1^KO^ mice to dextran sodium sulfate (DSS)-induced colitis.** WT (n = 37) and Nox1^KO^ (n = 30) mice were treated with 4% DSS in the drinking water or water alone (Ctrl) for the indicated time. (**A**) Representative AB/PAS-stained sections of the distal colon exhibited identical susceptibility to DSS despite the higher number of goblet cells in Nox1^KO^ mice than in WT. (**B**) Mouse body weight changes during DSS treatment are expressed as means ± sem. (**C**) Clinical disease activity index (DAI) score during 4% DSS administration was assessed, including weight loss, stool consistency, occult blood positivity, and gross rectal bleeding. (**D**) Cumulative histopathology score included the mucosal thickening, presence of inflammatory cells, general destruction of the architecture, loss of goblet cells. Statistics: Kruskal-Wallis non-parametric analysis, Dunn's multiple comparison test, NS, not significant.
(TIF)

Figure S8 **Susceptibility of 2,4,6-trinitrobenzenesulfonic acid (TNBS)-treated WT and Nox1^KO^ mice to severe colonic inflammation.** WT (n = 15) and Nox1^KO^ (n = 15) mice received an enema containing TNBS for 1 day. Controls (Ctrl) received ethanol enemas alone. (**A**) Representative AB/PAS-stained sections of the distal colon: the susceptibility to TNBS was similar in both mouse genotypes. (**B**) Mouse body weight changes during TNBS treatment are expressed as means ± sem. (**C**) Clinical disease activity index (DAI) score was assessed as in Fig. S8. (**D**) Cumulative histopathology score included the presence of inflammatory cells, general destruction of the architecture, ulcers. Statistics: Kruskal-Wallis non-parametric analysis, Dunn's multiple comparison test, NS, not significant.
(TIF)

Figure S9 **Susceptibility of WT and Nox1^KO^ mice to tunicamycin (TM) treatment.** WT (n = 5) and Nox1^KO^ (n = 5) mice received intraperitoneally 2 µg/g TM or its vehicle (Ctrl) and were sacrificed 24 h later. (**A**) Representative AB/PAS-stained sections of the distal colon: a more severe inflammation is observed in Nox1^KO^ mice than in WT mice. Note that the extensive focal crypt epithelial destruction, immune cell infiltrate, and loss of goblet cells are more pronounced in Nox1^KO^ mice than in WT mice. (**B**) Mouse body weight changes after TM treatment. (**C**) Clinical disease activity index (DAI) score was assessed as in Fig. S8. (**D**) Cumulative histopathology score was calculated in the proximal, median, and distal colon and included the presence of inflammatory cells, general destruction of the architecture, loss of goblet cells, ulcers. Statistics: *p*-values for Kruskal-Wallis non-parametric analysis are shown, Dunn's multiple comparison test, *p<0.05, **p<0.01, ***p<0.001.
(TIF)

Figure S10 **Expression of ER stress markers.** (**A**) The mRNA levels of spliced (*XBP-1s*) *XBP-1* form, *GRP78*, *GRP94*, *EDEM1, ATF4,* and *GADD34* in the distal colon of 3–4-week old WT (n = 10), Nox1^KO^ (n = 10), IL10^KO^ (n = 10), and IL10/Nox1^dKO^ (n = 10) mice were determined by qPCR and normalized to β-actin with the mean ratio of the WT group corrected to 1. Statistics: box plots show median, quartiles, and range; *p*-values for Kruskal-Wallis non-parametric analysis are shown, Dunn’s multiple comparison test versus WT, *p<0.05, **p<0.01, NS, not significant. (**B**) Crypt sections of the distal colon of 4-week old WT (n = 5), Nox1^KO^ (n = 5), IL10^KO^ (n = 5), and IL10/Nox1^dKO^ (n = 5) mice showing the immunohistochemical detection of ATF6α (left panel) and GRP78 (right panel). Note that ATF6α and GRP78 proteins are essentially expressed in the epithelial cells. (**C**) Representative immunoblot analysis of P-eIF2α (Ser^51^) and total eIF2α protein expression in the distal colon of WT (n = 5) and Nox1^KO^ (n = 5) mice treated or not (Ctrl) with 2 µg/kg tunicamycin (TM). β-actin was used as loading control. The P-eIF2α/eIF2α ratio was quantified and densitometric analyses are shown. *P*-values for Kruskal-Wallis non-parametric analysis are shown.
(TIF)

Figure S11 **(A) Concentrations of IL-1β, TNF-β, IL-17A, IFN-β, and IL-6 in the distal colonic explant supernatants of vehicle- (Vh, n = 8) or salubrinal (Sal, n = 10)-treated IL10/Nox1^dKO^ mice aged 6–7 weeks.** Statistics: box plots show median, quartiles, and range; *P*-values for Mann-Whitney U-test analysis are shown. (**B**) Representative immunohistological analysis of Foxp3^+^ cells in vehicle- (Vh, n = 5) or salubrinal (Sal, n = 5)-treated IL10/Nox1^dKO^ mice aged 6–7 weeks. (**C**) T_reg_ (CD4^+^ CD25^+^ Foxp3^+^) cell count in the spleen of vehicle- (Vh, n = 10) or salubrinal (Sal, n = 10)-treated IL10/Nox1^dKO^ mice aged 6–7 weeks expressed as a total lymphocyte percentage in the spleen. Statistics: box plots show median, quartiles, and range; *P*-values for Mann-Whitney U-test analysis are shown.
(TIF)

Table S1 Summary of the clinical characteristics of patients with UC and controls.
(TIF)
