# Supplementary figures and images for: Combined NADPH Oxidase 1 and Interleukin 10 Deficiency Induces Chronic Endoplasmic Reticulum Stress and Causes Ulcerative Colitis-Like Disease in Mice
Source: PLoS One. 2014 Jul 9;9(7):e101669. doi: 10.1371/journal.pone.0101669 (PMC4090121; doi:10.1371/journal.pone.0101669)

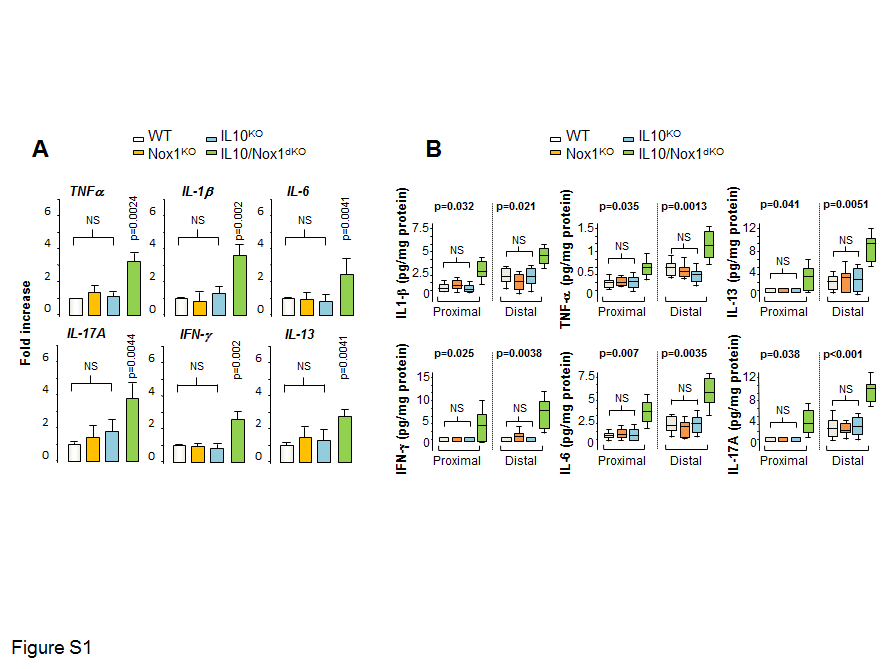

Supplement: Figure S1 — Cytokine expression and leukocyte composition in IL10/Nox1dKO mice. (A) Quantitative reverse transcriptase-PCR array was performed on the distal colonic sections of 7-week old WT (n = 5), Nox1KO (n = 5), IL10KO (n = 5), and IL10/Nox1dKO (n = 5) mice. Cytokine mRNA levels were normalized to GAPDH and expressed as relative fold change to the mean expression in WT mice. P-values for Kruskal-Wallis non-parametric analysis are shown; Dunn’s multiple comparison test vs. WT, NS, not significant. (B) Concentrations of different cytokines in supernatants from colonic lysates of 12-week old WT (n = 7), Nox1KO (n = 8), IL10KO (n = 8), and IL10/Nox1dKO (n = 10) mice. Statistics: box plots show median, quartiles, and range; p-values for Kruskal-Wallis non-parametric analysis are shown, Dunn's multiple comparison test vs. WT, NS, not significant. (TIF) [file pone.0101669.s001.tif]

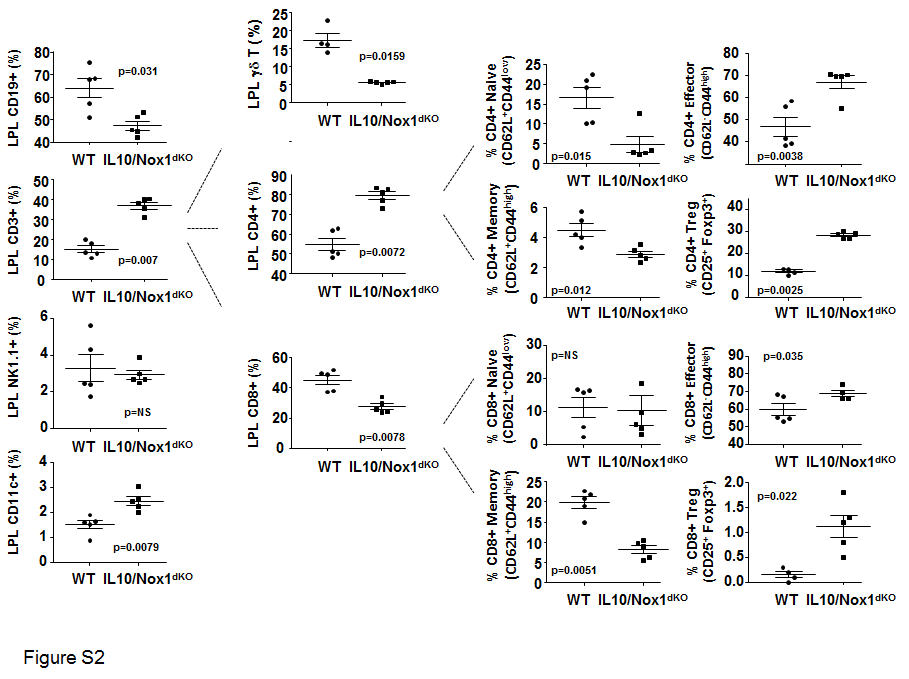

Supplement: Figure S2 — Altered lamina propria leukocyte composition in IL10/Nox1dKO mice. Lamina propria mononuclear cells from the colon of WT (n = 5) and IL10/Nox1dKO (n = 5) mice were stained for indicated markers and analyzed by flow cytometry, and expressed as a proportion of cells from the total live gate. Statistics: p-values for Mann-Whitney U-test are shown, NS; not significant. (TIF) [file pone.0101669.s002.tif]

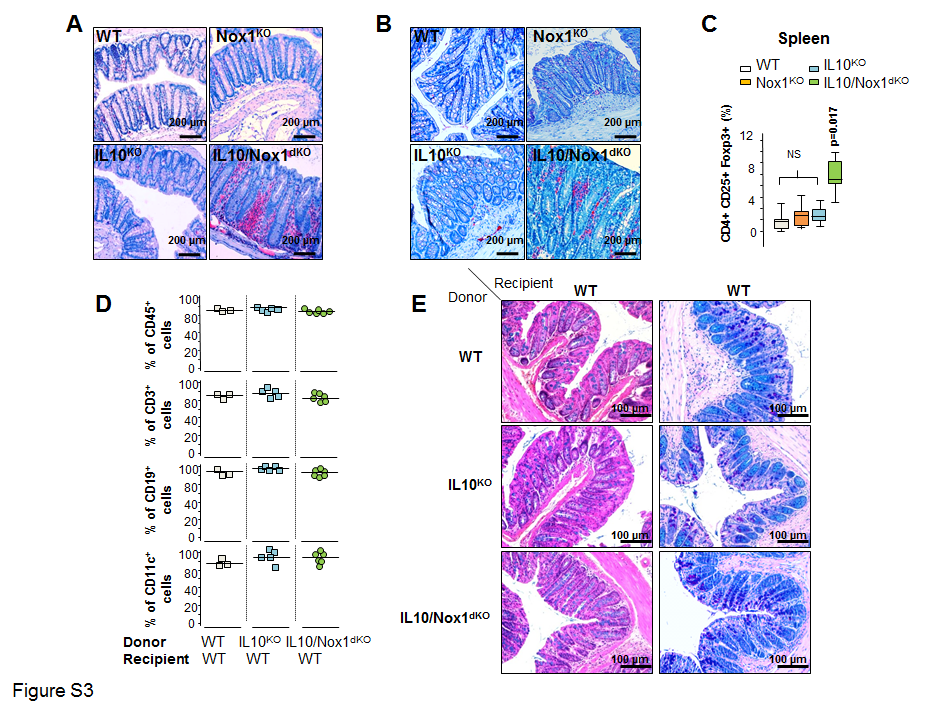

Supplement: Figure S3 — (A) Representative immunohistological analysis of CD3+ cells from distal colonic sections of 7-week old WT, Nox1KO, IL10KO, and IL10/Nox1dKO mice (n = 5/group). (B) Representative immunohistological analysis of Foxp3+ cells from distal colonic sections of 7-week old WT, Nox1KO, IL10KO, and IL10/Nox1dKO mice (n = 5/group). (C) Treg (CD4+ CD25+ Foxp3+) cell count in 7-week old IL10/Nox1dKO mice expressed as a total lymphocyte percentage in the spleen. Statistics: box plots show median, quartiles, and range; p-values for Kruskal-Wallis non-parametric analysis are shown, Dunn's multiple comparison test vs. WT, NS, not significant. (D) Bone marrow stem cells were isolated from WT, IL10KO or IL10/Nox1dKO CD45.2/Ly5.2 mice (4–6-week old) and injected intravenously into WT CD45.1/Ly5.1 lethally-irradiated recipients. Mice were studied 16 weeks after transplantation and the chimerism was assessed by flow cytometry using the Ly5.1 and Ly5.2 markers. Mononuclear cells were stained for CD45, CD3, CD19 or CD11c then analyzed by flow cytometry (individual points are shown). (E) Representative H&E- (left panels) and AB/PAS (right panels)-stained distal colonic sections of recipient WT mice reconstituted with WT, IL10KO or IL10/Nox1dKO bone marrow show normal colonic morphology and goblet cells. (TIF) [file pone.0101669.s003.tif]

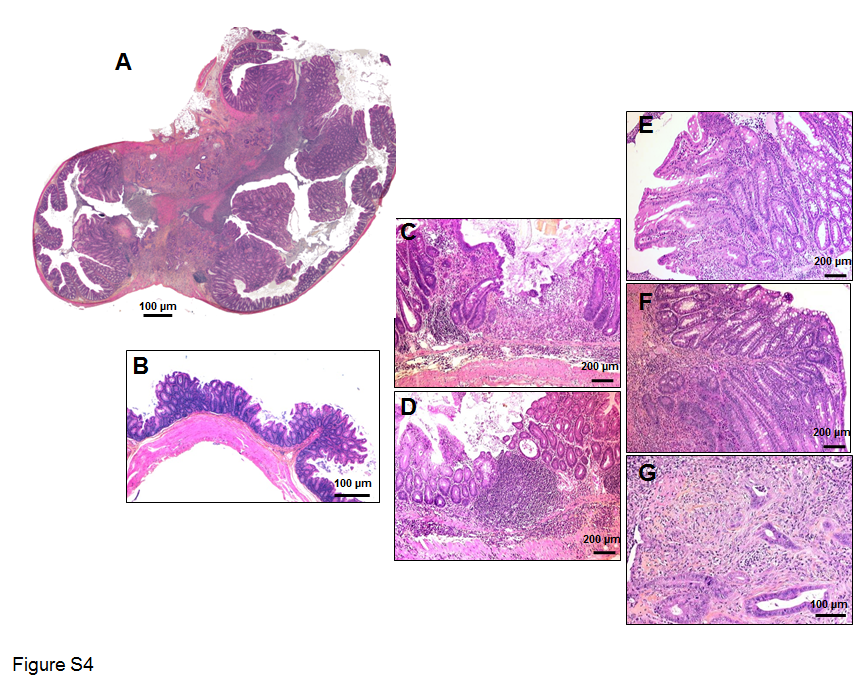

Supplement: Figure S4 — Natural history of spontaneous colitis-associated cancer in 8-month old IL10/Nox1dKO mice. (A) Histopathological whole-mount view of Swiss-roll showing dysplasia and multifocal cancer lesions developed in the colon (B) Histopathological image of dysplasia-associated lesion or mass (DALM). (C) Ulceration and basal plasmocytosis. (D) Crypt abscesses and plasmocytosis. (E) Low-grade dysplasia. (F) High-grade dysplasia. (G) Invasive adenocarcinoma occurring in the submucosa. (TIF) [file pone.0101669.s004.tif]

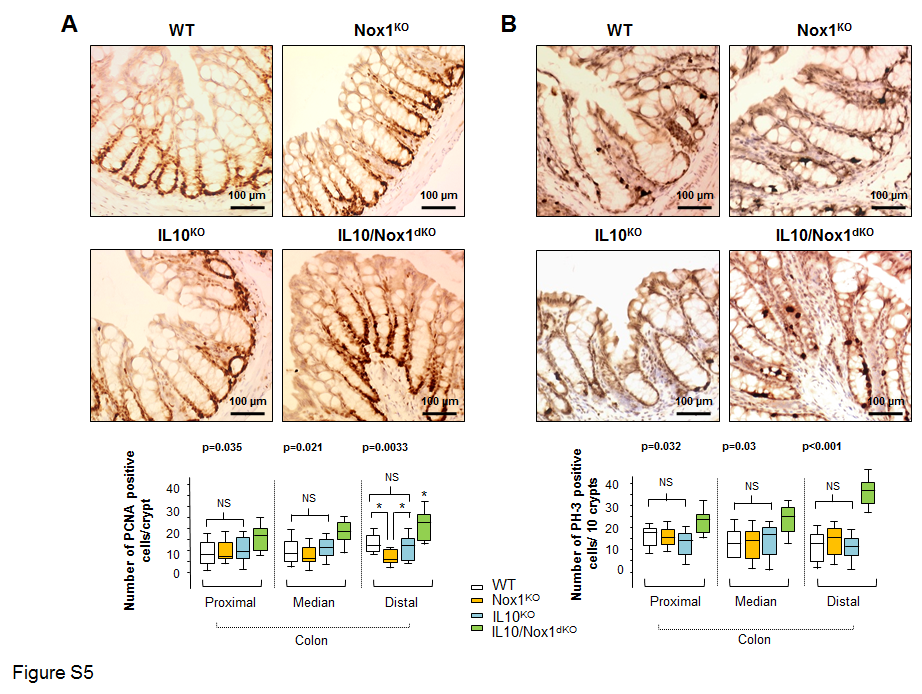

Supplement: Figure S5 — Colonic crypt proliferation is increased in IL10/Nox1dKO mice. Immunohistochemical analysis of the distal colonic sections of 7-week old WT (n = 5), Nox1KO (n = 5), IL10KO (n = 5), and IL10/Nox1dKO (n = 5) mice stained with antibodies against the proliferating antigens (A) PCNA and (B) phospho-histone-3 (PH-3). The number of PCNA+ and PH3+ nuclei was counted in 10 and 50 consecutive crypts from proximal, median, and distal colon, respectively. Statistics: box plots show median, quartiles, and range; p-values for Kruskal-Wallis non-parametric analysis are shown, Dunn's multiple comparison test vs. WT, *p<0.05, NS, not significant. (TIF) [file pone.0101669.s005.tif]

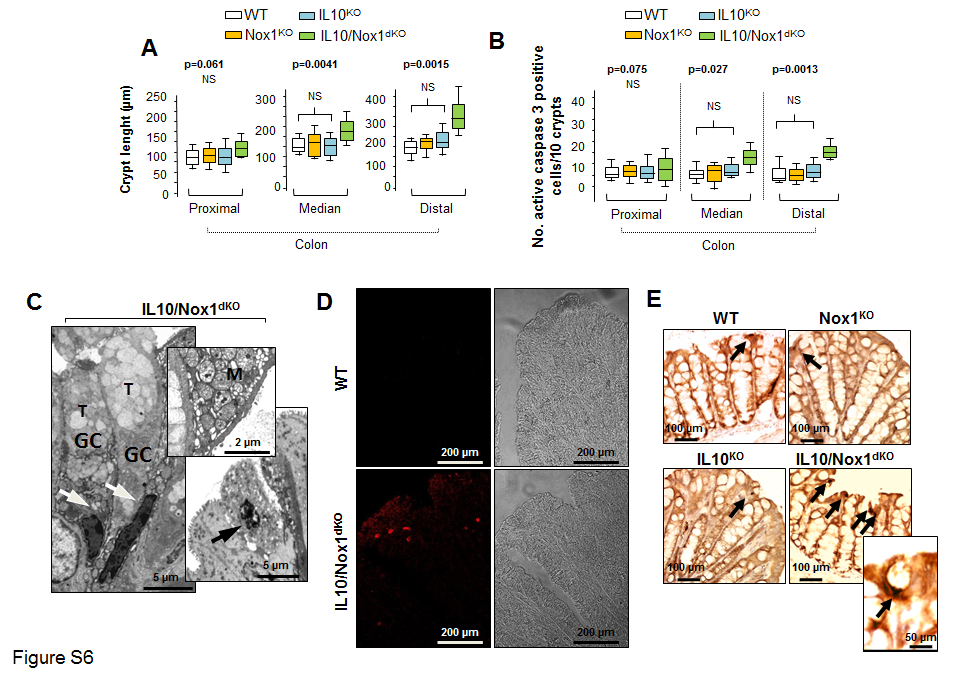

Supplement: Figure S6 — Increased proliferation and apoptosis in the colonic crypts of IL10/Nox1dKO mice. (A) Length of proximal, median, and distal colonic crypts in 6–7-week old WT (n = 10), Nox1KO (n = 10), IL10KO (n = 15), and IL10/Nox1dKO (n = 15) mice. Statistics: box plots show median, quartiles, and range; p-values for Kruskal-Wallis non-parametric analysis are shown, Dunn's multiple comparison test vs. WT, NS, not significant. (B) The number of active caspase 3 positive cells was counted in 10 consecutive crypts from proximal, median, and distal colon of 7-week old WT (n = 10), Nox1KO (n = 10), IL10KO (n = 15), and IL10/Nox1dKO (n = 15) mice. Statistics: box plots show median, quartiles, and range; p-values for Kruskal-Wallis non-parametric analysis are shown, Dunn's multiple comparison test vs. WT, NS, not significant. (C) Transmission electron micrographs of the distal colon of 7-week old IL10/Nox1dKO mice (n = 5) reveal reduced size of goblet cell (GC) thecae (T), pycnotic GC nuclei with irregular edge (white arrows), altered mitochondria (M) and cytoplasm vacuolization, apoptotic fragments and vacuole containing condensed GC debris (black arrows). (D) Confocal microscopy of colonic sections of WT and IL10/Nox1dKO mice stained with antibody against active caspase 3 (red). Original magnification (x40). (E) Representative immunohistological analysis of active caspase 3 in distal colonic sections of 6-week old WT (n = 5), Nox1KO (n = 5), IL10KO (n = 10), and IL10/Nox1dKO (n = 10) mice. Magnification of the micrographs shows increased immunostaining of active caspase 3 in IL10/Nox1dKO mouse GC. (TIF) [file pone.0101669.s006.tif]

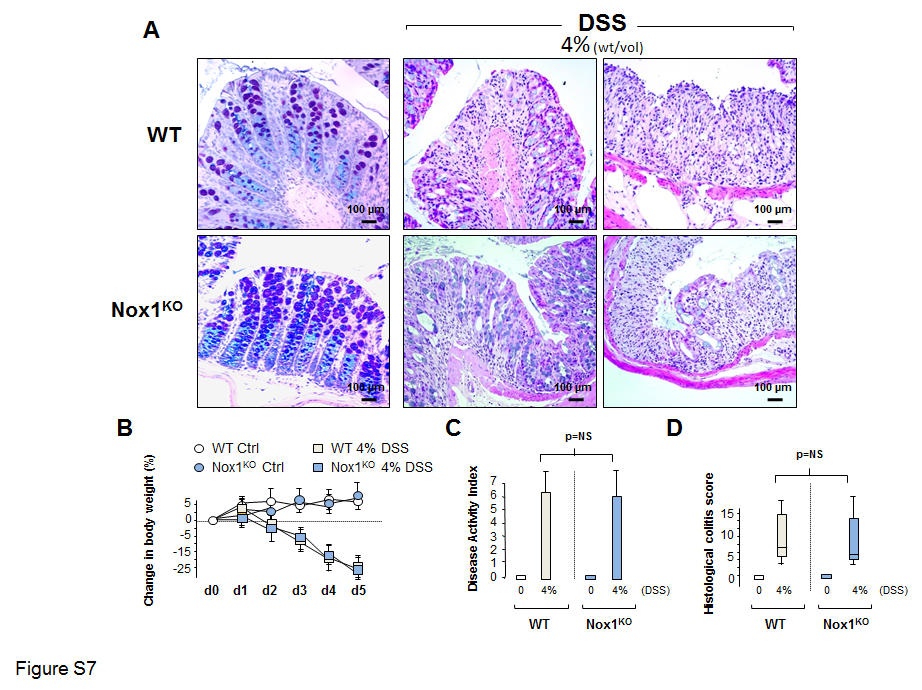

Supplement: Figure S7 — Susceptibility of WT and Nox1KO mice to dextran sodium sulfate (DSS)-induced colitis. WT (n = 37) and Nox1KO (n = 30) mice were treated with 4% DSS in the drinking water or water alone (Ctrl) for the indicated time. (A) Representative AB/PAS-stained sections of the distal colon exhibited identical susceptibility to DSS despite the higher number of goblet cells in Nox1KO mice than in WT. (B) Mouse body weight changes during DSS treatment are expressed as means ± sem. (C) Clinical disease activity index (DAI) score during 4% DSS administration was assessed, including weight loss, stool consistency, occult blood positivity, and gross rectal bleeding. (D) Cumulative histopathology score included the mucosal thickening, presence of inflammatory cells, general destruction of the architecture, loss of goblet cells. Statistics: Kruskal-Wallis non-parametric analysis, Dunn's multiple comparison test, NS, not significant. (TIF) [file pone.0101669.s007.tif]

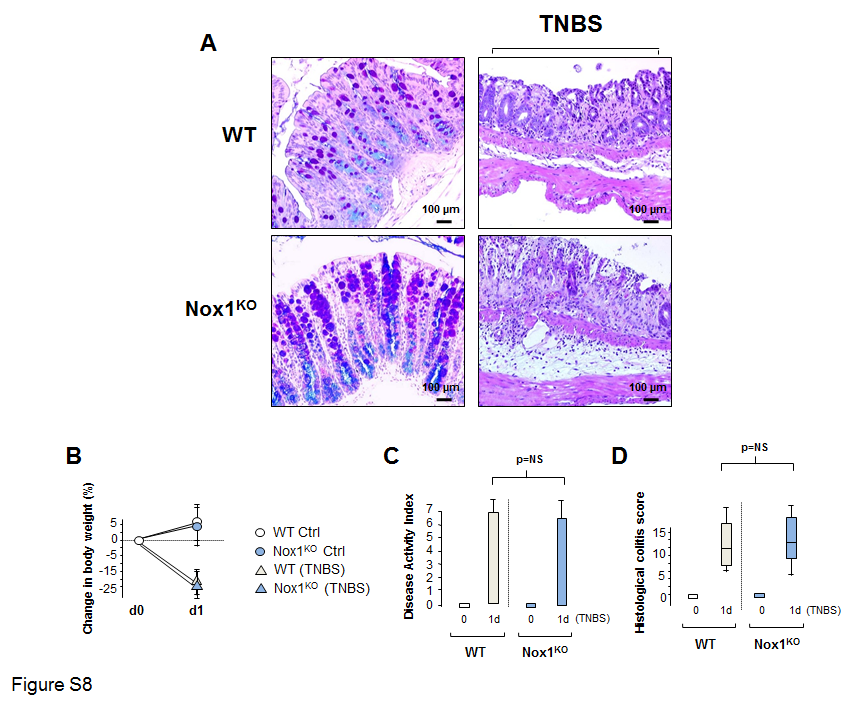

Supplement: Figure S8 — Susceptibility of 2,4,6-trinitrobenzenesulfonic acid (TNBS)-treated WT and Nox1KO mice to severe colonic inflammation. WT (n = 15) and Nox1KO (n = 15) mice received an enema containing TNBS for 1 day. Controls (Ctrl) received ethanol enemas alone. (A) Representative AB/PAS-stained sections of the distal colon: the susceptibility to TNBS was similar in both mouse genotypes. (B) Mouse body weight changes during TNBS treatment are expressed as means ± sem. (C) Clinical disease activity index (DAI) score was assessed as in Fig. S8. (D) Cumulative histopathology score included the presence of inflammatory cells, general destruction of the architecture, ulcers. Statistics: Kruskal-Wallis non-parametric analysis, Dunn's multiple comparison test, NS, not significant. (TIF) [file pone.0101669.s008.tif]

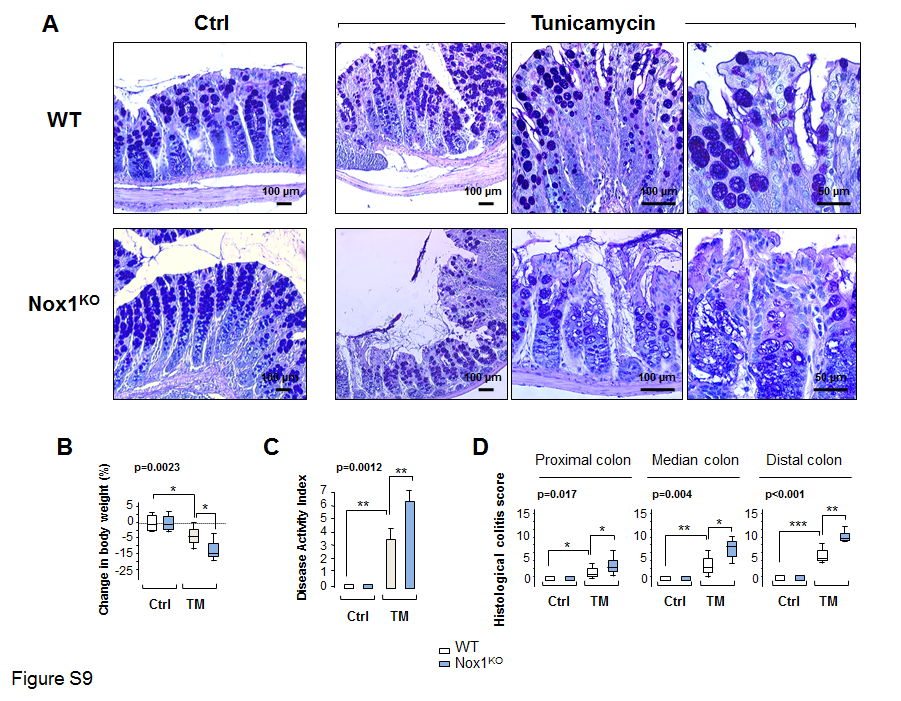

Supplement: Figure S9 — Susceptibility of WT and Nox1KO mice to tunicamycin (TM) treatment. WT (n = 5) and Nox1KO (n = 5) mice received intraperitoneally 2 µg/g TM or its vehicle (Ctrl) and were sacrificed 24 h later. (A) Representative AB/PAS-stained sections of the distal colon: a more severe inflammation is observed in Nox1KO mice than in WT mice. Note that the extensive focal crypt epithelial destruction, immune cell infiltrate, and loss of goblet cells are more pronounced in Nox1KO mice than in WT mice. (B) Mouse body weight changes after TM treatment. (C) Clinical disease activity index (DAI) score was assessed as in Fig. S8. (D) Cumulative histopathology score was calculated in the proximal, median, and distal colon and included the presence of inflammatory cells, general destruction of the architecture, loss of goblet cells, ulcers. Statistics: p-values for Kruskal-Wallis non-parametric analysis are shown, Dunn's multiple comparison test, *p<0.05, **p<0.01, ***p<0.001. (TIF) [file pone.0101669.s009.tif]

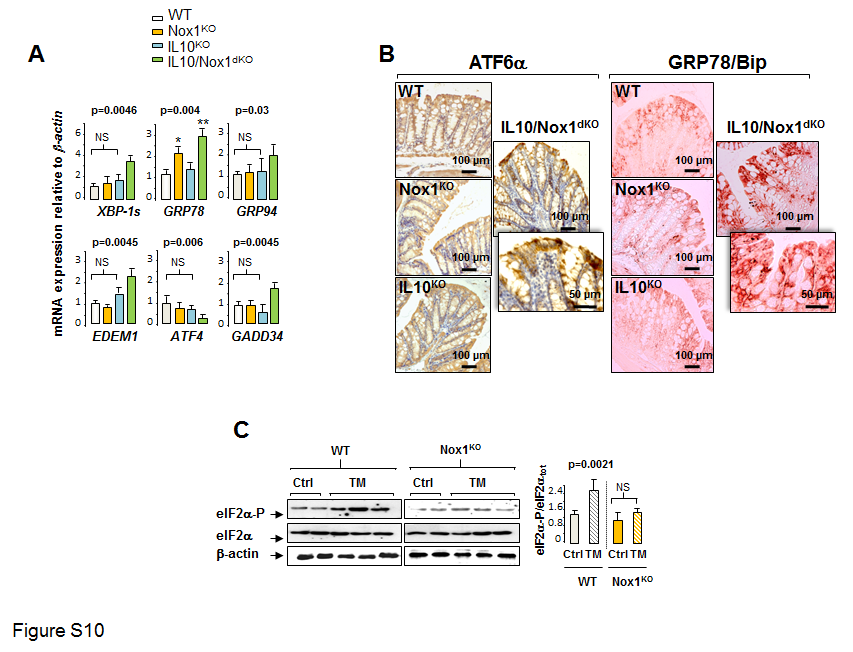

Supplement: Figure S10 — Expression of ER stress markers. (A) The mRNA levels of spliced (XBP-1s) XBP-1 form, GRP78, GRP94, EDEM1, ATF4, and GADD34 in the distal colon of 3–4-week old WT (n = 10), Nox1KO (n = 10), IL10KO (n = 10), and IL10/Nox1dKO (n = 10) mice were determined by qPCR and normalized to β-actin with the mean ratio of the WT group corrected to 1. Statistics: box plots show median, quartiles, and range; p-values for Kruskal-Wallis non-parametric analysis are shown, Dunn’s multiple comparison test versus WT, *p<0.05, **p<0.01, NS, not significant. (B) Crypt sections of the distal colon of 4-week old WT (n = 5), Nox1KO (n = 5), IL10KO (n = 5), and IL10/Nox1dKO (n = 5) mice showing the immunohistochemical detection of ATF6α (left panel) and GRP78 (right panel). Note that ATF6α and GRP78 proteins are essentially expressed in the epithelial cells. (C) Representative immunoblot analysis of P-eIF2α (Ser51) and total eIF2α protein expression in the distal colon of WT (n = 5) and Nox1KO (n = 5) mice treated or not (Ctrl) with 2 µg/kg tunicamycin (TM). β-actin was used as loading control. The P-eIF2α/eIF2α ratio was quantified and densitometric analyses are shown. P-values for Kruskal-Wallis non-parametric analysis are shown. (TIF) [file pone.0101669.s010.tif]

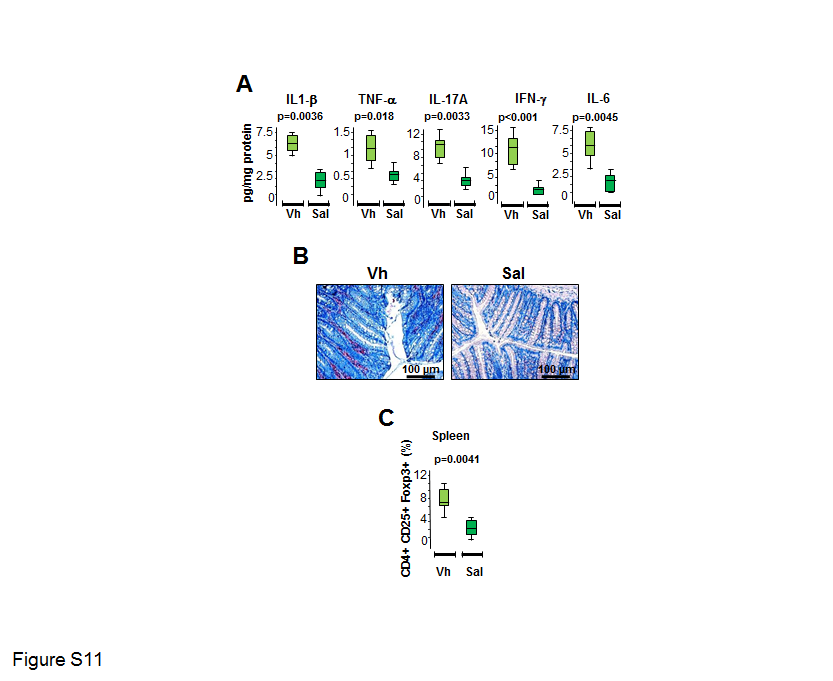

Supplement: Figure S11 — (A) Concentrations of IL-1β, TNF-β, IL-17A, IFN-β, and IL-6 in the distal colonic explant supernatants of vehicle- (Vh, n = 8) or salubrinal (Sal, n = 10)-treated IL10/Nox1dKO mice aged 6–7 weeks. Statistics: box plots show median, quartiles, and range; P-values for Mann-Whitney U-test analysis are shown. (B) Representative immunohistological analysis of Foxp3+ cells in vehicle- (Vh, n = 5) or salubrinal (Sal, n = 5)-treated IL10/Nox1dKO mice aged 6–7 weeks. (C) Treg (CD4+ CD25+ Foxp3+) cell count in the spleen of vehicle- (Vh, n = 10) or salubrinal (Sal, n = 10)-treated IL10/Nox1dKO mice aged 6–7 weeks expressed as a total lymphocyte percentage in the spleen. Statistics: box plots show median, quartiles, and range; P-values for Mann-Whitney U-test analysis are shown. (TIF) [file pone.0101669.s011.tif]

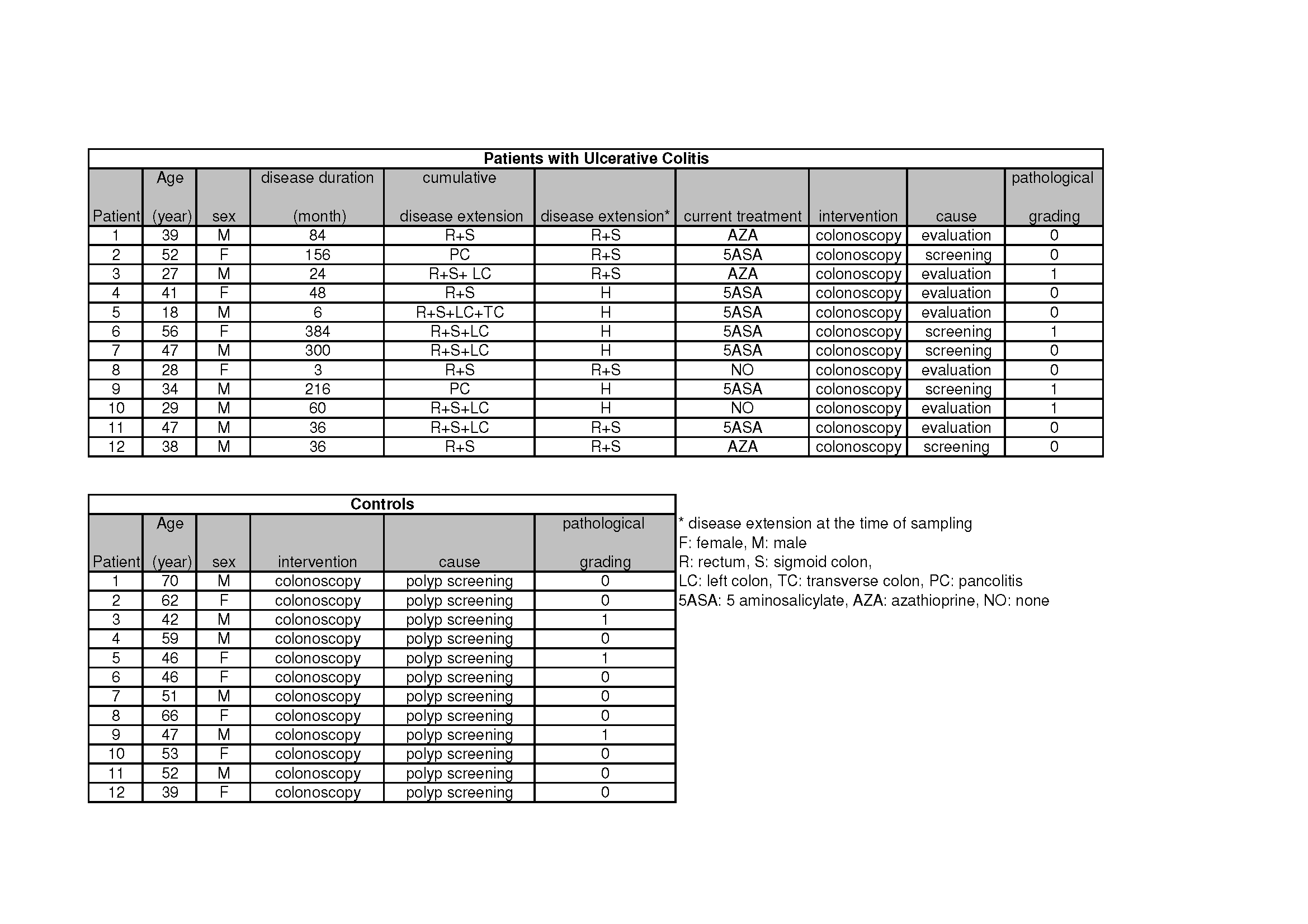

Supplement: Table S1 — Summary of the clinical characteristics of patients with UC and controls. (TIF) [file pone.0101669.s012.tif]
